# Divergent Evolution of Lanthipeptide Stereochemistry

**DOI:** 10.1021/acschembio.2c00492

**Published:** 2022-08-24

**Authors:** Raymond Sarksian, Wilfred A. van der Donk

**Affiliations:** † Department of Chemistry and Howard Hughes Medical Institute, University of Illinois at Urbana-Champaign, Urbana, Illinois 61822, United States; ‡ Carl R. Woese Institute for Genomic Biology, University of Illinois at Urbana-Champaign, Urbana, Illinois 61822, United States

## Abstract

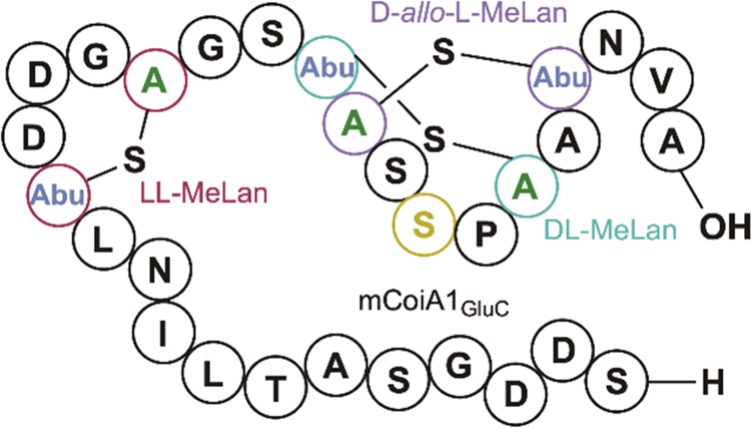

The three-dimensional structure of natural products is critical for their biological activities and, as such, enzymes have evolved that specifically generate active stereoisomers. Lanthipeptides are post-translationally modified peptidic natural products that contain macrocyclic thioethers featuring lanthionine (Lan) and/or methyllanthionine (MeLan) residues with defined stereochemistry. In this report, we compare two class I lanthipeptide biosynthetic gene clusters (BGCs), *coi* and *olv*, that represent two families of lanthipeptide gene clusters found in Actinobacteria. The precursor peptides and BGCs are quite similar with genes encoding a dehydratase, cyclase, and methyltransferase (MT). We illustrate that the precursor peptide CoiA1 is converted by these enzymes into a polymacrocyclic product, mCoiA1, that contains an analogous ring pattern to the previously characterized post-translationally modified OlvA peptide (mOlvA). However, a clear distinction between the two BGCs is an additional Thr-glutamyl lyase (GL) domain that is fused to the MT, CoiS_A_, which results in divergence of the product stereochemistry for the *coi* BGC. Two out of three MeLan rings of mCoiA1 contain different stereochemistry than the corresponding residues in mOlvA, with the most notable difference being a rare d-*allo*-l-MeLan residue, the formation of which is guided by CoiS_A_. This study illustrates how nature utilizes a distinct GL to control natural product stereochemistry in lanthipeptide biosynthesis.

## Introduction

Natural products typically recognize their targets with exquisite affinity and selectivity.^1^ In the course of their evolution, the structures of these molecules have been optimized to bind to the usually chiral environments of biological targets. This high level of recognition is often achieved by the rich stereochemistry of natural products, which have made them privileged ligands.^1^ In this study, we report the discovery of two lanthipeptides that have very similar ring patterns but in which the stereochemistry of two of the three macrocycles differs. We show that the acquisition of one new enzyme has driven the divergence of the two compound groups and that this new activity has resulted in coevolution of other enzymes in the pathway.

**Figure 1:**
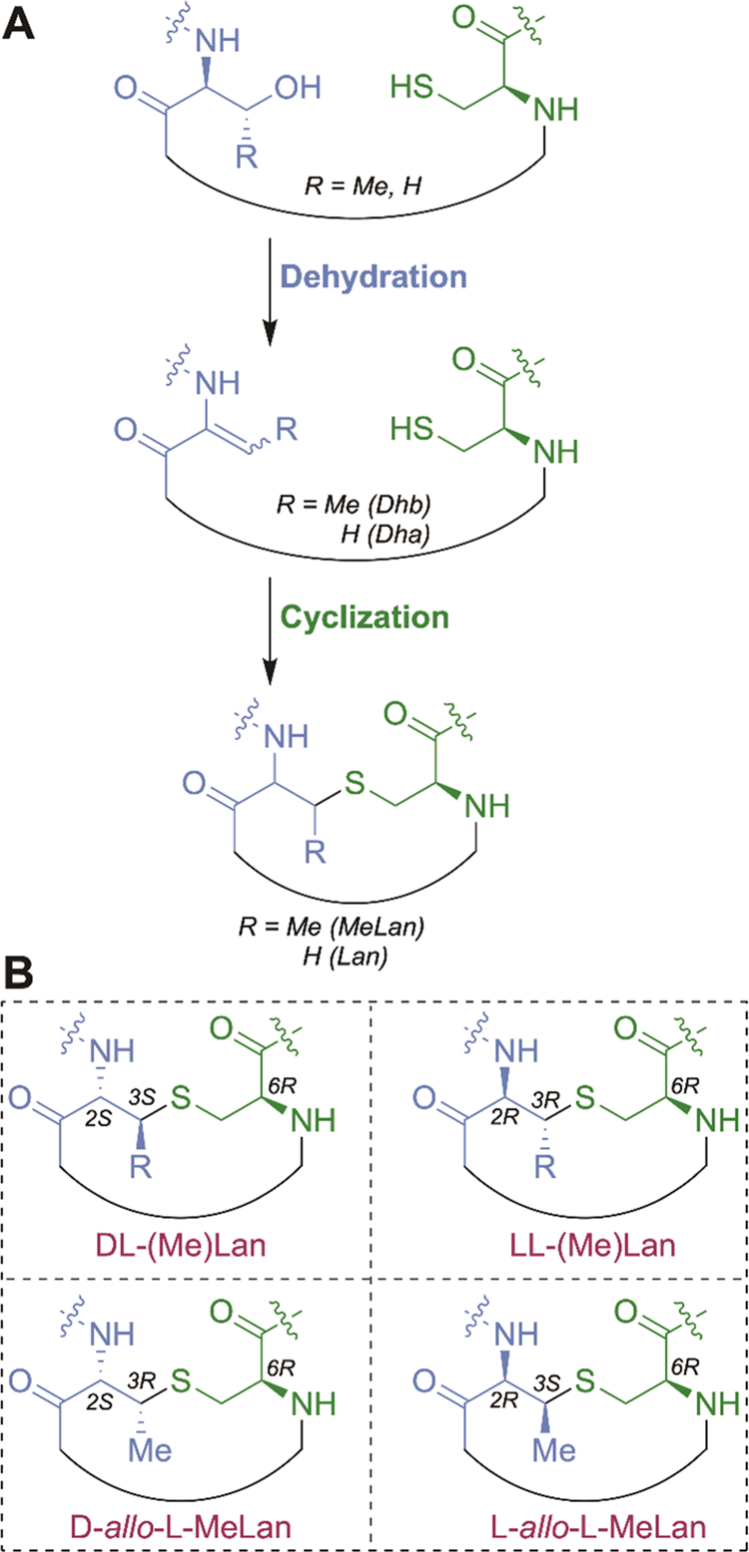
(A) Maturation of lanthipeptides proceeds through dehydration of Ser/Thr residues to generate the corresponding dehydroamino acids dehydroalanine and dehydrobutyrine (Dha, Dhb). Cyclization of Cys side chains onto Dha/Dhb yields (Me)Lan. (B) Four possible MeLan diastereomers can be generated as shown.

Lanthipeptides represent one of the largest classes of ribosomally synthesized and posttranslationally modified peptides (RiPPs).^2−4^ They exhibit a wide range of activities, including antimicrobial, antiviral, morphogenetic, and antifungal,^3,5−10^ and are defined by the presence of lanthionine (Lan) or methyllanthionine (MeLan) residues (Figure 1). Maturation of lanthipeptides features the dehydration of Ser/Thr residues followed by subsequent Michael-type addition of a Cys thiol onto the dehydroamino acids to form (Me)Lan (Figure 1).^2−4^

For class I lanthipeptides, a lanthipeptide dehydratase LanB first catalyzes the transesterification of a glutamate group from glutamyl-tRNA^Glu^ to the side chain of Ser/Thr.^11−14^ An elimination reaction of the glutamylated intermediate generates dehydroalanine (Dha) from Ser or dehydrobutyrine (Dhb) from Thr (Figure 1A). A LanC cyclase then catalyzes the formation of thioether rings by the addition of a Cys thiol to the dehydroamino acid intermediates.^15,16^

The stereochemical configuration of (Me)Lan residues has been shown to be important for the biological activities of lanthipeptides.^17,18^ Three MeLan diastereomers have been discovered to date, (*2S,3S,6R*)-, (*2R*,*3R*,*6R*)-, and (*2S*,*3R*,*6R*)-MeLan, hereafter referred to as dl-, ll-, and d-*allo*-l-MeLan (Figure 1B).^4,19−25^ Both dl- and ll-MeLan are believed to form through the *anti*-elimination of Thr residues to yield (*Z*)-Dhb residues followed by an *anti*-addition of Cys across the (*Z*)-Dhb.^4^ Facial selectivity of the cyclization event dictates whether dl- or ll-MeLan is formed from the (*Z*)-Dhb.^20^d-*allo*-l-MeLan was recently reported for the morphogenetic class I lanthipeptide SapT.^24^d-*allo*-l-MeLan is thought to be formed through *syn*-elimination of glutamylated Thr residues followed by subsequent *anti*-addition of Cys across the (*E*)-Dhb.^24^ The SapT biosynthetic gene cluster (BGC) features a split dehydratase made up of SptB_a_ and SptB_b_ that carries out dehydration. SptB_a_ catalyzes glutamylation of Ser/Thr residues, and SptB_b_ is a glutamyl lyase (GL) that catalyzes *syn*-elimination.^24^

**Figure 2:**
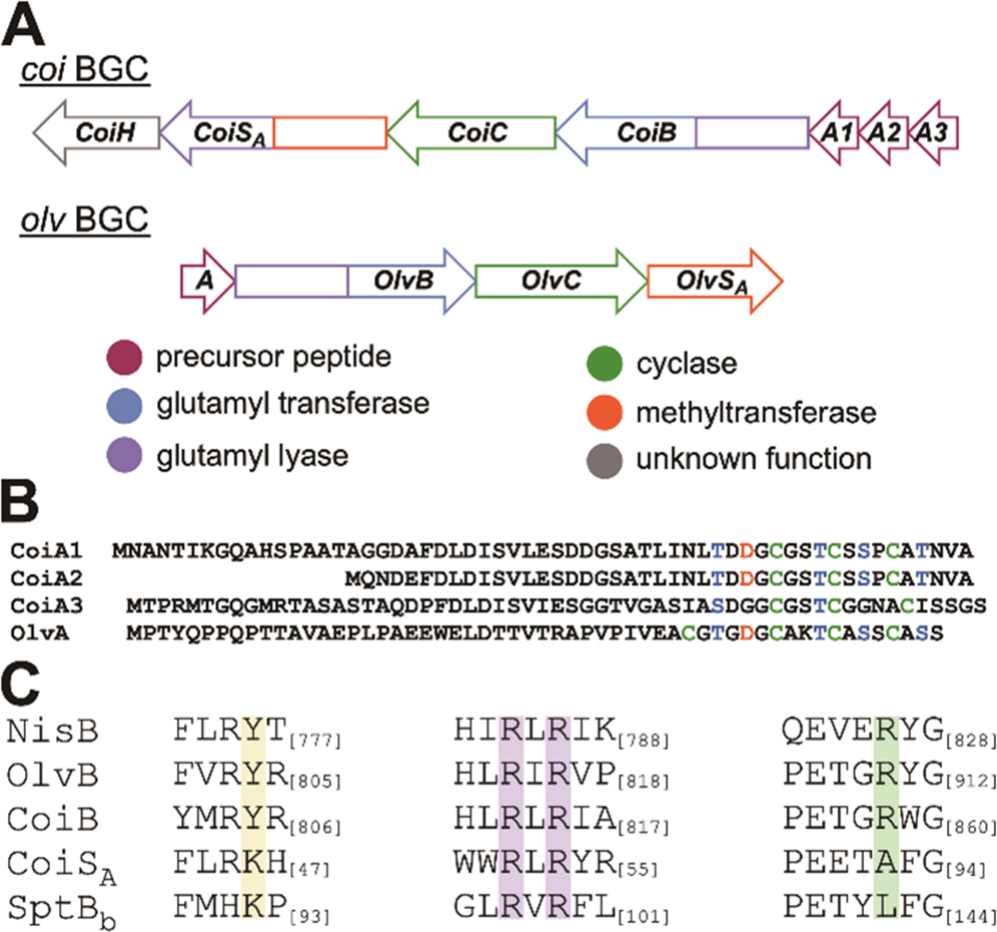
(A) Comparison of *coi* and *olv* class I lanthipeptide BGCs. The *coi* BGC encodes an additional GL and protein of unknown function when compared to the *olv* BGC. (B) Sequence alignment of the precursor peptides encoded in both BGCs. Residues that are known to be modified in OlvA are colored. (C) Sequence alignment of GL domains illustrates that the additional GL domain in CoiS_A_ is similar to SptB_b_.

In this study, we compare the *coi* BGC from *Streptomyces coelicolor* A3(2) with the *olv* BGC from *Streptomyces olivaceus* NRRL B-3009 (Figure 2A). These BGCs are representative examples of two groups of gene clusters in Actinobacteria (Figure 2A), with only the *olv* BGC previously investigated in depth.^26^ Both BGCs encode a canonical class I dehydratase (CoiB and OlvB) and cyclase (CoiC and OlvC), and an *O*-methyltransferase (MT) that is the most widespread auxiliary enzyme in class I lanthipeptide BGCs.^26−28^ The sequences of their precursor peptides are also quite similar (Figure 2B). The ring pattern and stereochemistry of the (Me)Lan residues of the *olv* product have been determined by nuclear magnetic resonance (NMR) spectroscopy and comparison with synthetic standards.^26^ The *coi* BGC mainly differs from the *olv* BGC in that the MT CoiS_A_ has a fused GL domain and encodes a protein of unknown function CoiH.^24,26^

The presence of two GLs in the *coi* BGC is unusual. The GL domains in both CoiB and CoiS_A_ contain conserved Arg residues that are important for the recognition of the γ-carboxylate of the glutamylated peptide intermediate in lanthipeptide dehydratases (Figure 2C).^11−14^ However, only CoiB contains the catalytic His base and Arg residue that are critical for glutamate elimination activity in canonical *anti*-GLs (Figures 2C and S1).^11−14^ The GL domain in CoiS_A_ in contrast contains similar putative active site residues as SptB_b_.^24^

Here, we investigate the regioselectivity of the two distinct GL domains in CoiB and CoiS_A_ (Figure 2). Both enzymes catalyze glutamate elimination; however, they are proposed to generate two different Dhb isomers during the maturation of CoiA1.^24^ We demonstrate that the 3-fold dehydrated and cyclized product, mCoiA1, contains three different MeLan diastereomers in a ring pattern that is very similar to the *olv* product. However, elucidation of the stereochemical configuration for each MeLan residue showed that two of the three residues have different stereochemistry compared to that found in the *olv* product. Furthermore, our data show that CoiC catalyzes cyclization with both (*Z*)- and (*E*)-Dhb residues but only when these isomers are at their native location, suggesting coevolution of the cyclase with the product stereochemistry. These findings provide intriguing insights into the divergent evolution of two widespread natural products by acquisition (or deletion) of a GL that results in different stereochemistry.

## Results

### Heterologous Production and Characterization of mCoiA1

**Figure 3:**
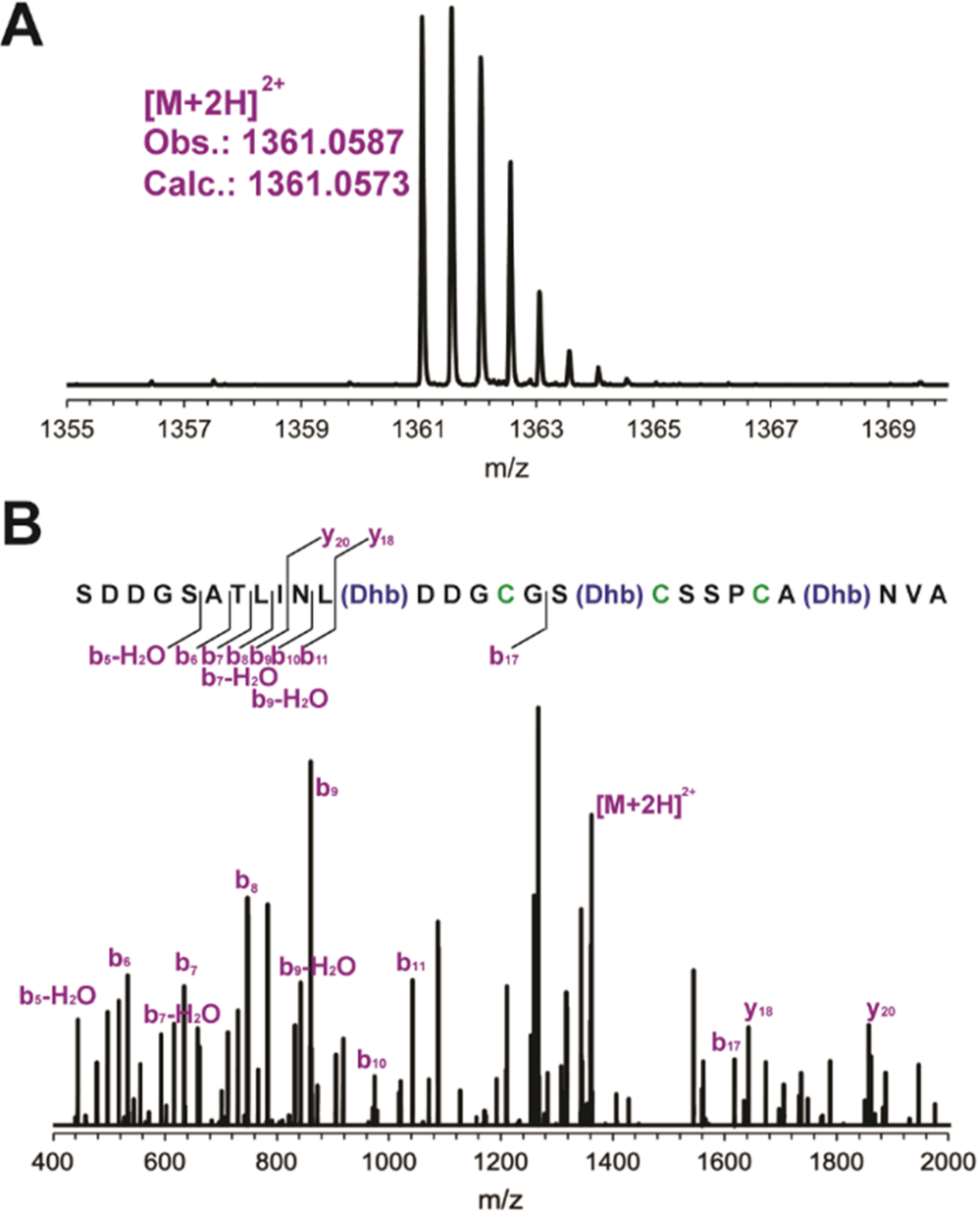
Liquid chromatography-MS (LC-MS) analysis of the GluC digestion product of mCoiA1 (mCoiA1_GluC_). (A) High-resolution electrospray ionization-MS (ESI-MS) analysis. (B) Tandem ESI-MS analysis. Fragmentation results are consistent with an N-terminal MeLan and two C-terminal overlapping MeLan.

The *coi* cluster is present in the genome of one of the most widely studied strains of *Streptomyces*, *S. coelicolor* A3(2),^29−32^ but its product has not been previously observed despite considerable genome mining studies.^33−37^ Therefore, in this study, we used heterologous expression to investigate its product. CoiA1 was previously shown to undergo 3-fold dehydration and cyclization to yield mCoiA1 when coexpressed with CoiB, the elimination domain of CoiS_A_ (CoiS_A(ED)_), and CoiC in *Escherichia coli*.^24^ In this work, the coexpressed product was isolated and treated with endoproteinase GluC (Figure 3). High-resolution mass spectrometry (HRMS) analysis confirmed that the product was dehydrated three times (Figure 3A). Tandem MS of the GluC-digested peptide suggests that it contains an N-terminal MeLan ring and two C-terminal overlapping MeLan rings (Figure 3B, Table S3). This ring pattern is similar to that determined by tandem MS and NMR spectroscopy for the *olv* product,^26^ consistent with the conservation of the positions of the Ser/Thr and Cys residues in the CoiA1 and OlvA precursor peptides (Figure 2B).

### Glutamate Elimination Activity

**Figure 4:**
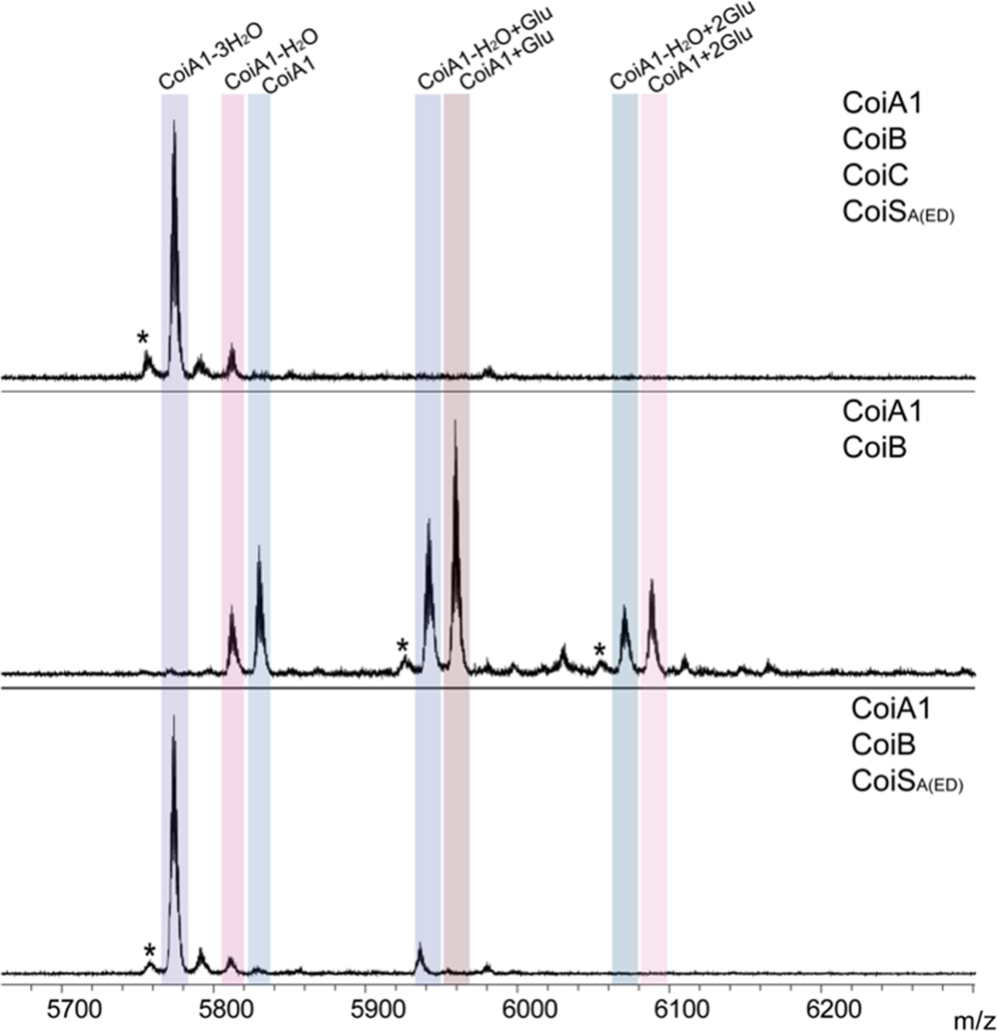
Matrix-assisted laser desorption ionization time-of-flight (MALDI-TOF) MS analysis of coexpression experiments in *E. coli*. Coexpressed proteins are listed on each panel. Asterisks indicate deamination products that are commonly generated in MALDI-TOF mass spectra at these masses.

Next, we performed experiments to gain insight into the elimination activity of both CoiB and CoiS_A(ED)_. Coexpression of CoiA1 with CoiB and CoiC led to at most one dehydration along with intermediates that were glutamylated once or twice (Figure 4). This finding suggests that the C-terminal lyase domain of CoiB was only able to perform one elimination of the Ser/Thr residues that were glutamylated by the N-terminal domain of CoiB. In contrast, when CoiA1 was coexpressed with CoiS_A(ED)_, CoiC, and the CoiB-H994A mutant, in which the lyase activity of the C-terminal domain of CoiB was inactivated but the glutamylation activity was retained,^12,14^ a 3-fold dehydrated product was observed (Figure S2). Thus, CoiS_A(ED)_ was able to eliminate glutamate at all three Thr residues.

An *N*-ethylmaleimide (NEM) alkylation assay^38^ to test for the presence of free Cys suggested that the product peptide of the latter experiment was not fully cyclized (Figure S3), implying that the 3-fold dehydrated peptide generated by CoiS_A(ED)_ is not a competent substrate for CoiC. These results show that both CoiB and CoiS_A(ED)_ are required for correct dehydration and cyclization of CoiA1. Finally, we investigated whether CoiC is necessary to obtain a 3-fold dehydrated product. In the case of some lanthipeptides such as microbisporicin A1,^13^ the lanthipeptide cyclase is required to obtain full dehydration since select (Me)Lan rings must form prior to the next dehydration event. Coexpression of CoiA1 with CoiB and CoiS_A(ED)_ resulted in a 3-fold dehydrated product (Figure 4). Therefore, CoiC and the formation of (Me)Lan rings are not necessary for combined CoiB and CoiS_A(ED)_ activity.

### Stereochemical Analysis of MeLan Residues

**Figure 5:**
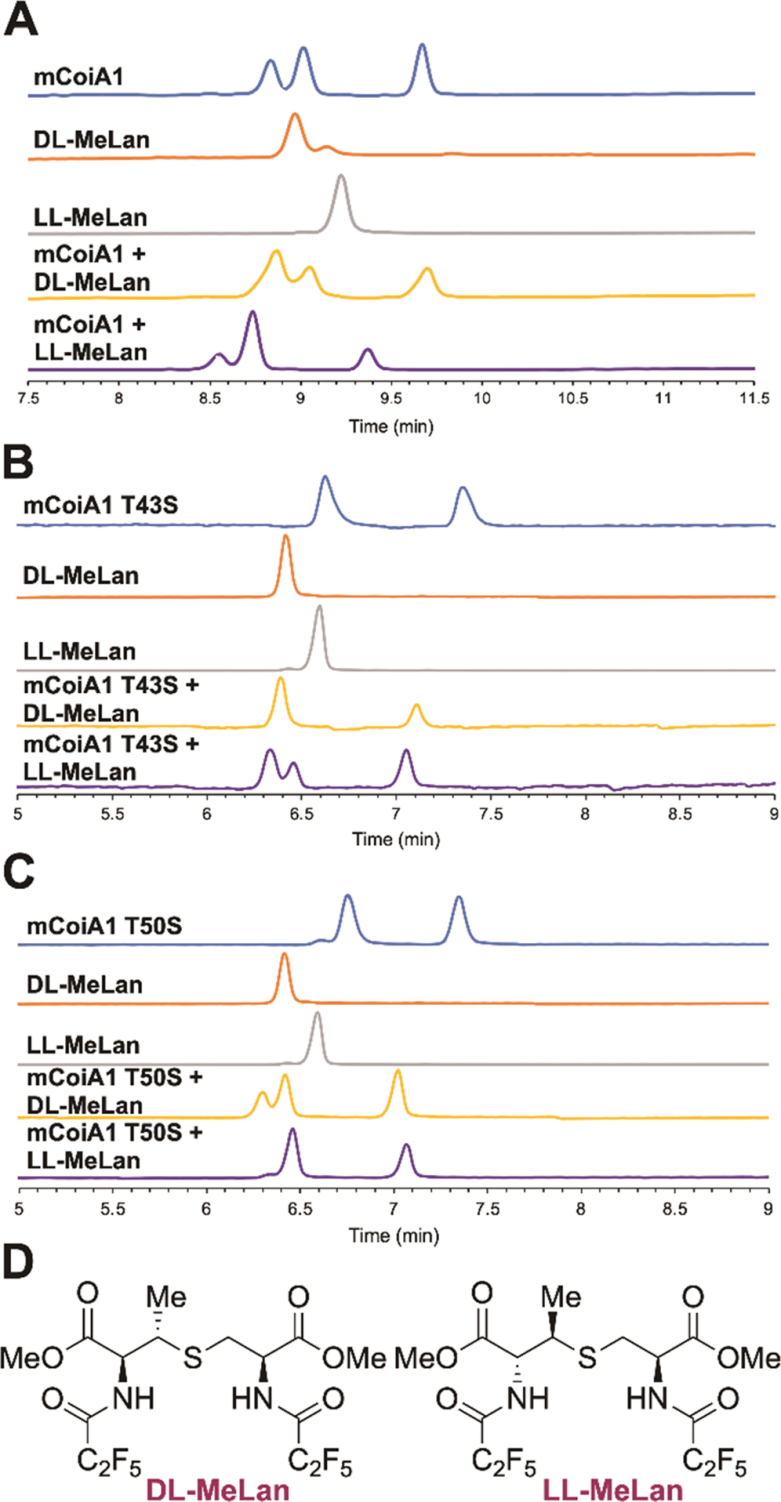
Gas chromatography-MS (GC-MS) analysis of derivatized MeLan residues (*m*/*z* = 379) obtained from mCoiA1 mutant peptides using a chiral stationary phase. Coinjections with stereochemically pure derivatized dl- and ll-MeLan confirm the presence of dl-MeLan in mCoiA1-T43S and ll-MeLan in mCoiA1-T50S. (A) Derivatized MeLan from WT mCoiA1 (top), synthetic standards, and coinjections of the products of mCoiA1 with the synthetic standards. (B) Derivatized MeLan from mCoiA1-T43S (top), synthetic standards, and coinjections of the products of mCoiA1-T43S with the synthetic standards. (C) Derivatized MeLan from mCoiA1-T50S (top), synthetic standards, and coinjections of the products of mCoiA1-T50S with the synthetic standards. (D) Structures of derivatized dl- and ll-MeLan standards.

mCoiA1 was previously demonstrated to contain dl-, ll-, and d-*allo*-l-MeLan residues (Figure 5A).^24^ Assignment of the stereochemistry to specific rings was not reported. Since mCoiA1 only contains MeLan rings, Thr residues involved in ring formation were individually mutated to Ser residues to determine the stereochemistry for each MeLan ring.^25^ If successful, this approach would remove a single MeLan residue (by conversion to a Lan residue) in each variant and thus allow assignment of stereochemistry to that MeLan.

CoiA1-T43S, CoiA1-T50S, and CoiA1-T57S variants were generated by site-directed mutagenesis and coexpressed with CoiB, CoiS_A(ED)_, and CoiC followed by isolation of the products by Ni-affinity chromatography. mCoiA1-T43S and mCoiA1-T50S underwent 3-fold dehydration and were unreactive toward NEM, suggesting that the peptides were cyclized (Figure S4). HRMS and tandem MS confirmed that the cyclization patterns of these two mutant peptides were not altered from WT mCoiA1 (Figures S5 and S6). In contrast, mCoiA1-T57S was obtained in multiple dehydration states and was not fully processed (Figure S4). Hence, only mCoiA1-T43S and -T50S were hydrolyzed in acid and the resulting amino acids were derivatized for stereochemical analysis.

Stereochemical analysis was performed by gas chromatography-mass spectrometry (GC-MS) on a chiral stationary phase and comparison to authentic dl- and ll-MeLan standards.^25,39,40^ mCoiA1-T43S contained two MeLan, as anticipated (Figure 5B). Coinjections with stereochemically pure dl- and ll-MeLan confirmed the presence of dl-MeLan and absence of ll-MeLan (Figure 5B). Therefore, WT mCoiA1 must contain an N-terminal ll-MeLan ring derived from Thr43. Using the same approach, mCoiA1-T50S also revealed peaks in the GC-MS corresponding to two MeLan (Figure 5C). Coinjections confirmed one of the MeLan peaks to consist of MeLan with the ll-configuration and dl-MeLan was absent in the sample. By the same logic, WT mCoiA1 must contain a dl-MeLan ring derived from Thr50.

The second-eluting MeLan peak from mCoiA1-T43S and mCoiA1-T50S that did not match either dl- or ll-MeLan was anticipated to be the rare *allo* isomer as previously detected in WT mCoiA1.^24^ The two possible *allo*-MeLan stereoisomers were previously shown to be inseparable by GC-MS.^24^ Hydrolysis of mCoiA1-T43S and mCoiA1-T50S and derivatization of the amino acids with Marfey’s reagent, *N*_α_-(2,4-dinitro-5-fluorophenyl)-l-alaninamide (l-FDAA), followed by comparison to d-*allo*-d/l-MeLan and l-*allo*-d/l-MeLan standards^24^ confirmed the presence of d-*allo*-d/l-MeLan in both peptides by liquid chromatography-mass spectrometry (Figure S7). mCoiA1 was next demonstrated to contain d-*allo*-l-MeLan and not d-*allo*-d-MeLan by isolation of MeLan and reductive desulfurization, which would form d-Ala from d-*allo*-d-MeLan or l-Ala from d-*allo*-l-MeLan (Figure S8).^9^ The desulfurization product consisted of l-Ala (Figure S8) confirming the presence of d-*allo*-l-MeLan. Because this isomer is also seen in both mCoiA1-T43S and mCoiA1-T50S, the MeLan derived from Thr57 in mCoiA1 must be d-*allo*-l-MeLan. Based on the sequence similarity of CoiS_A(ED)_ with SptB_b_, the rare d-*allo*-l-MeLan is generated through the involvement of CoiS_A(ED)_. The poor conversion of CoiA1-T57S suggests that mutation of Thr57 to Ser is not well tolerated by CoiS_A(ED)_ and may imply that some *syn*-GLs are specific for Thr.

### CoiS_A(ED)_ Mutational Analysis

Mutational analysis was next performed on CoiS_A(ED)_ to decipher the importance of putative active site residues. Based on sequence analysis, CoiB is very similar to the nisin dehydratase NisB and related class I lanthipeptide dehydratases that catalyze *anti*-elimination (Figure S1).^11−14^ GLs that catalyze *anti*-elimination have been well characterized both biochemically and structurally. In contrast, *syn*-GLs have only recently been discovered, and their putative active sites diverge from *anti*-GLs (Figures 2C and S1). CoiS_A(ED)_, SptB_b_, and related homologs contain a highly conserved Lys residue that when mutated in CoiS_A(ED)_ (CoiS_A(ED)_-K46A) resulted in the accumulation of glutamylated peptides implying its importance for glutamate elimination.^24^

**Figure 6:**
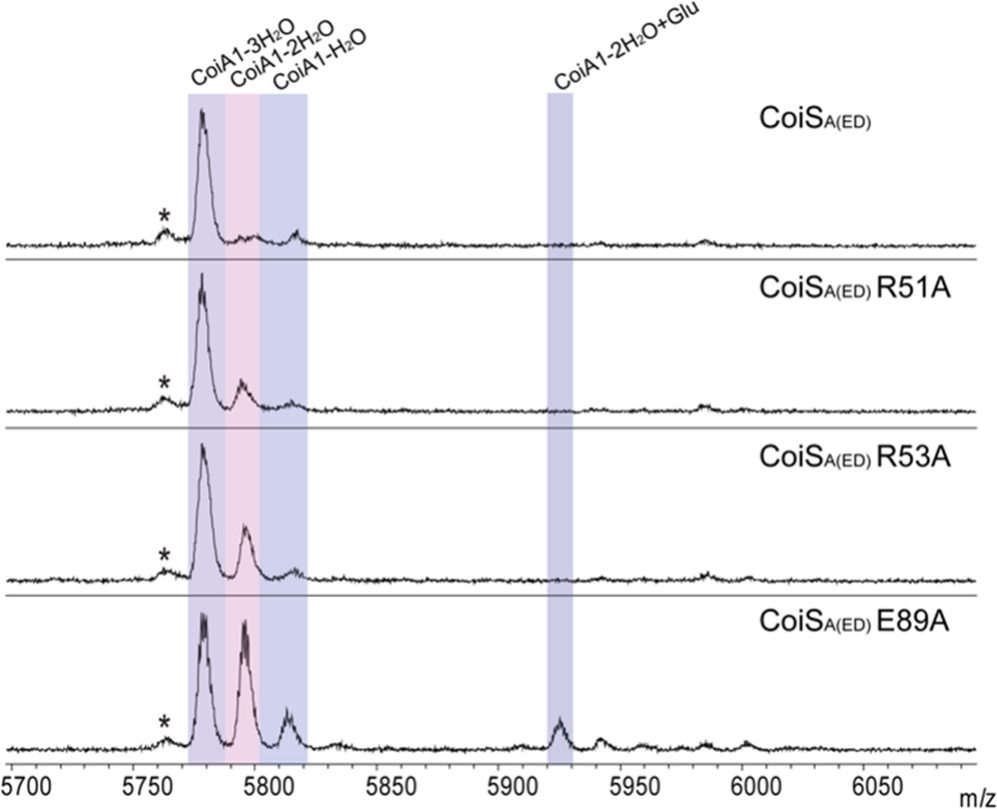
MALDI-TOF MS analysis of CoiA1 coexpression with CoiB, CoiC, and CoiS_A(ED)_ WT or CoiS_A(ED)_ mutants. Asterisks indicate deamination products that are commonly generated in MALDI-TOF mass spectra at these masses.

In addition to the differences observed between *syn*- and *anti*-GLs, some key sequence similarities are also found. In NisB, Arg784 and Arg786 bind to the γ-carboxylate of the glutamylated peptide intermediate and are critical for elimination activity.^12−14^ These residues are also conserved in CoiS_A(ED)_, and Ala mutants were generated to determine the importance of activity. Both CoiS_A(ED)_-R51A and -R53A mutants were coexpressed with CoiA1, CoiB, and CoiC. For both mutants, a 3-fold dehydrated product was obtained, along with a 2-fold dehydrated product (Figure 6). Therefore, in contrast to NisB, the Arg residues do not seem to be absolutely critical as elimination activity is not severely diminished. Finally, we generated CoiS_A(ED)_-E89A. This Glu is highly conserved across all class I lanthipeptide GLs (Figure S1; Glu823 in NisB), and based on a calculated structure of SptB_b_, the residue points toward the putative active site and may play a role in catalysis.^24^ Coexpression experiments of CoiA1, CoiB, and CoiC with the CoiS_A(ED)_-E89A mutant revealed that dehydration activity was altered, but a 3-fold dehydrated peptide was still generated (Figure 6). Thus, Glu89 is also not critical for catalysis by CoiS_A(ED)_.

### Bioinformatic Analysis and Comparison of *anti-* and *syn-*GLs

Two key differences exist between *anti*- and *syn*-GLs investigated thus far based on sequence and mutational analysis. A highly conserved Arg residue (Arg826 in NisB) is present in *anti*-GLs (Figure 2C) that interacts with the carbonyl oxygen of glutamylated Thr to lower the p*K*_a_ of the α-proton and facilitate elimination.^14^ For SptB_b_, CoiS_A(ED)_, and related homologs, this residue is replaced by a hydrophobic residue (Figure 2C). In addition, SptB_b_ and CoiS_A(ED)_ contain a highly conserved Lys residue that is important for elimination activity that is a Tyr in *anti*-GLs.

**Figure 7:**
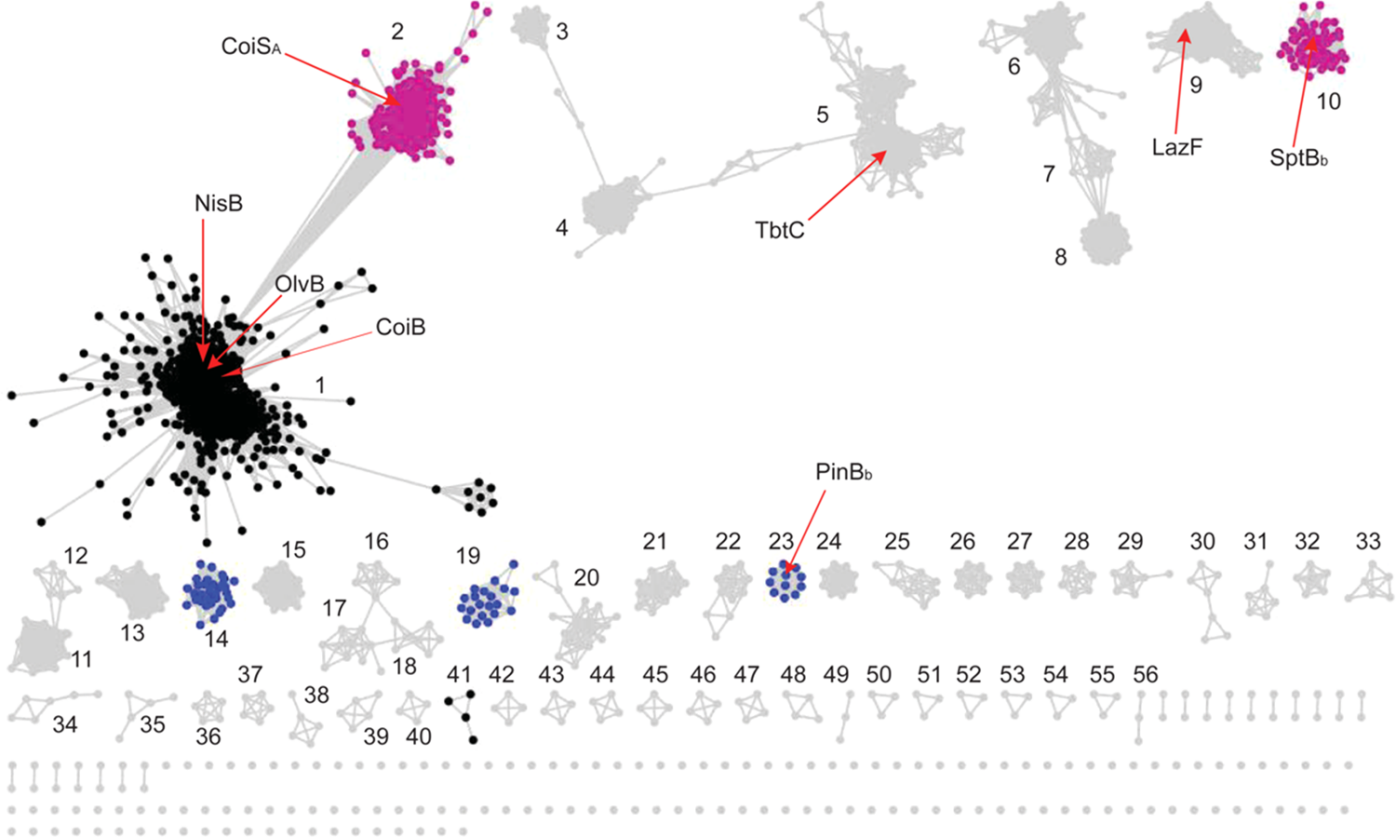
SSN analysis of GLs. All colored groups indicate GLs within class I lanthipeptide BGCs. Full-length LanB dehydratases that catalyze *anti*-elimination are shown in black, and *anti*-GLs within split LanB systems in blue. *Syn*-GLs that are part of a split LanB system that are fused to a methyltransferase or that are present as stand-alone proteins are depicted in purple. GLs within BGCs of other nonlanthipeptide or hybrid RiPPs are in gray. A select number of characterized GLs are labeled including the thiopeptide GLs TbtC and LazF. For brief summaries of the biosynthetic genes in each group, see Table S6. The cytoscape file for the SSN is provided as the Supporting Information.

We surveyed all GLs involved in the BGCs of lanthipeptides and related RiPPs using these sequence differences. A sequence-similarity network (SSN) for GLs was previously generated using the tools of the Enzyme Function Initiative (Figure 7).^41,42^ In this study, the genomic context of each group was inspected and the GLs in the class I lanthipeptide BGCs were selected for sequence alignments to determine whether they belong to *anti*-GLs or *syn*-GLs (Figure 7 and Table S6). This analysis suggested grouping of the GLs into three broad categories. The largest group of GLs is the full-length LanB proteins that are all predicted to catalyze *anti*-elimination (Figures 7, black, and S1). Both CoiB and OlvB are found within this group. Smaller groups of *anti*-GLs are part of split LanB systems with dedicated glutamyl transferases and glutamate lyases (Figures 7, blue, and S1). Consistent with a previous study,^24^ a significant portion of the network also revealed *syn*-GLs as (1) part of a split LanB system, (2) fused to a methyltransferase domain such as CoiS_A_, or (3) present as an additional stand-alone GL domain, with the latter two in BGCs that also contain a full-length dehydratase (Figure 7, purple). All other groups containing more than two members were inspected and found to be part of BGCs of other RiPPs such as thiopeptides (Figure 7, gray, and Table S6). Analysis of GLs within these BGCs suggests that the associated GLs are similar to *anti*-GLs and are likely generating (*Z*)-Dhb and/or Dha residues as either intermediates and/or in the final products. Hence, the occurence of *syn*-GLs seems to be limited in the currently sequenced genomes to class I lanthipeptide BGCs. The SSN also provided the opportunity to assess whether (*E*)-Dhb and/or *allo*-MeLan isomers are present in any previously reported class I lanthipeptides for which stereochemistry has generally not been determined. The active site residues of GLs involved in the biosynthesis of these previously characterized family members were analyzed (Figure S9). These GLs were found to be very similar to *anti*-GLs suggesting that (*E*)-Dhbs and/or *allo*-MeLan isomers are likely not present in these lanthipeptides.

## Discussion

**Figure 8:**
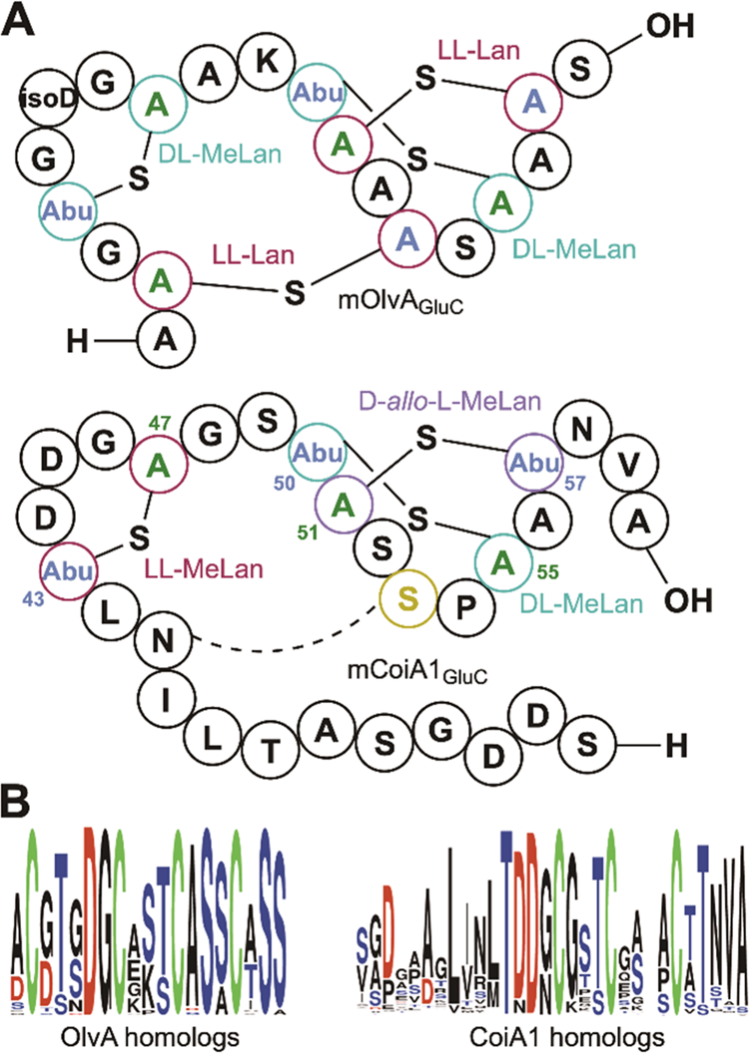
(A) Comparison of the structures of GluC-digested mCoiA1 and mOlvA to highlight differences and similarities in ring patterns and stereochemistry. Abu, 2-aminobutyric acid. The N-terminal Lan ring in mOlvA is absent in mCoiA1, although the corresponding Ser residue (gold) is present. (B) Sequence LOGO made using orthologs of the core peptide region of OlvA and CoiA1 identified by BLAST analysis^45^ to highlight conserved residues (OlvA total sequences = 43, CoiA1 total sequences = 301).

The overall structure of mCoiA1 in terms of the stereochemical configuration of (Me)Lan residues is the most complex of any lanthipeptide characterized thus far. mCoiA1 contains an N-terminal ll-MeLan ring and overlapping C-terminal dl- and d-*allo*-l-MeLan rings (Figure 8A). Except for an additional Lan in mOlvA, mCoiA1 and mOlvA contain (Me)Lan rings at equivalent positions. It is interesting to note that the Ser residue that is involved in this additional N-terminal Lan in OlvA is also present in CoiA1 but escapes dehydration. The corresponding Cys residue to generate the Lan ring is missing in CoiA1 and replaced by an Asn residue (Figures 2B and 8A).

Although the ring patterns of mOlvA and mCoiA1 are similar, the stereochemical configurations of two of the three (Me)Lan rings differ. In the C-terminal region of these peptides, mOlvA contains an ll-Lan, whereas mCoiA1 contains a rare d-*allo*-l-MeLan residue (Figure 8A). A critical distinction between the *coi* and *olv* BGCs is the presence of the additional GL domain in CoiS_A_, which is similar to SptB_b_ that was previously proposed to catalyze *syn*-elimination of glutamylated Thr to generate (*E*)-Dhb that leads to the observed d-*allo*-l-MeLan in SapT.^24^ The second deviation in stereochemistry is that mCoiA1 contains an N-terminal ll-MeLan ring where mOlvA contains a dl-MeLan ring at the equivalent position (Figure 8A). Unlike the change in stereochemistry at the C-terminus, which is accounted for by the presence of a gene encoding an additional *syn*-GL in the BGC, the origin of the change in the stereochemistry of the N-terminal MeLan is less clear. The conformational landscape of lanthipeptides has been shown to be important for the cyclization reaction.^43,44^ Therefore, one possibility is that the additional N-terminal ll-Lan ring in mOlvA is formed early during the biosynthetic process and that this ring conformationally biases the peptide toward forming an alternative diastereomer for the adjacent ring. Alternatively, the change in the stereochemistry of the C-terminal MeLan because of the recruitment of a new GL could in turn also influence the stereochemistry of the N-terminal ring if the d-*allo*-l-MeLan is formed early in the maturation process. Regardless of the molecular explanation of the change in stereochemistry, in all investigated examples, engineered changes in stereochemistry have led to the abolishment of the original bioactivity of the lanthipeptide.^17,18^ Therefore, it is likely that the differences in stereochemistry between the products of the widespread *coi* and *olv* gene cluster families have functional consequences.

Several examples have been reported wherein RiPP biosynthesis requires an obligate order of post-translational modifications.^3^ The investigation of the *coi* BGC provides another example of high coordination of the post-translational modification reactions. Based on the stereochemistry of the final product, it is likely that CoiB converts Thr43 and Thr50 into (*Z*)-Dhb residues, which are then the substrates for CoiC-catalyzed cyclization events that provide the ll- and dl-MeLan residues, respectively. The elimination domain of CoiS_A_ likely dehydrates Thr57 to (*E*)-Dhb, which CoiC then converts to d-*allo*-l-MeLan. When CoiS_A(ED)_ was coexpressed with CoiA1 and CoiC and a variant of CoiB that can still glutamylate but not eliminate, three dehydrations were still observed, but the cyclase was unable to form the three thioether macrocycles, presumably because the peptide now contained three (*E*)-Dhb residues. Thus, CoiC is only able to accept (*E*)-Dhb at position 57 and requires (*Z*)-Dhb at positions 43 and 50 for cyclization activity. The observation that CoiC is capable of cyclization of Cys51 onto (*E*)-Dhb57 but apparently cannot catalyze the addition of Cys47 and Cys55 to (*E*)-Dhb43 and 50, respectively, is suggestive of coevolution of CoiC with the appearance of CoiS_A_ in the pathway. Conversely, when only CoiB was coexpressed with CoiA1 and CoiC, the dehydration process stalled at a single dehydration and one or two glutamylations. These findings suggest that either the *syn*-elimination of Thr57 by CoiS_A(ED)_ is required for CoiB to complete its dehydration and/or that CoiC must first form the d-*allo*-l-MeLan for full CoiB activity. The data also suggest that after CoiS_A_/CoiC act on Thr57, glutamylation of Thr43 and Thr50 by the N-terminal domain of CoiB is followed by faster glutamate elimination by the GL domain of CoiB than by CoiS_A(ED)_. These findings therefore suggest a highly choreographed set of biosynthetic reactions to make a complex ring pattern with high fidelity.

Orthologs of the CoiA1 peptide are much more common than orthologs of the OlvA peptide in the currently sequenced genomes (Figure 8B, Tables S7 and S8). Whether the *coi*-like BGCs evolved from the *olv*-like clusters by recruitment of a new GL domain or by gene duplication or whether the *olv-*like BGCs lost the gene for the *syn*-GL is a difficult question. Based on the preponderance of *anti*-GLs in diverse RiPP BGCs (Figure 7), it is likely that the ancestral enzyme catalyzed *anti*-elimination, but this hypothesis cannot be unambiguously verified at present. What is clear is that during evolution in Actinobacteria, two distinct BGCs with a common origin diverged and that stereochemistry was very likely a key determining factor. Determining the function of the *coi* and *olv* BGC products and how stereochemistry may alter the biological activities of these compounds is therefore of great interest. Investigations to answer these questions will first need to determine the cleavage site between the leader and core peptide, which is hampered by the absence of any reports detecting the products of these BGCs in their native producing organisms and the absence of a protease in the BGC that could provide insight regarding the start position of the final product.

## Conclusions

The *coi* BGC is highly similar in architecture to the previously investigated *olv* BGC with a key distinction being the presence of an additional GL domain that is fused to the MT CoiS_A_. We illustrate that mCoiA1 has a similar ring pattern to mOlvA with one less Lan ring and that it has different stereochemistry for two out of the three MeLan rings. The most pronounced difference between the two products is the recently discovered rare d-*allo*-l-MeLan diastereomer that is the result of the additional GL domain of CoiS_A_. This study illustrates an example of divergent evolution driven by stereochemistry, which in turn is likely to be correlated to the function of the final products.

## Supporting Information Available

Glutamyl lyase SSN (ZIP)
Experimental procedures; extended experimental data and bioinformatic analysis; materials and methods; primers used in this study; observed and calculated *m*/*z* ratios for fragments of GluC-digested mCoiA1; NEM alkylation assay for CoiA1 co-expressed with CoiB-H994A, CoiC, and CoiS_A(ED)_; LC-MS analysis of the GluC digestion product of mCoiA1 and variants; description table of orthologs obtained from PSI-BLAST of OlvA (PDF)

## Accession Codes

NCBI protein accessions: CoiA1, WP_011031313.1; CoiB, WP_011031312.1; CoiC, WP_011031311.1; and CoiS_A_, WP_011031310.1.
