# Supplementary material for: Divergent Evolution of Lanthipeptide Stereochemistry
Source: ACS Chem Biol. 2022 Aug 24;17(9):2551–8. doi: 10.1021/acschembio.2c00492 (PMC9486935; doi:10.1021/acschembio.2c00492)
Supplement: Supplementary file 2 — cb2c00492_si_002.pdf [file cb2c00492_si_002.pdf]

Divergent Evolution of Lanthipeptide Stereochemistry

Supporting Information

Raymond Sarksian[1] and Wilfred A. van der Donk[1,2*]

[1] Department of Chemistry and Howard Hughes Medical Institute, University of Illinois at Urbana-Champaign, Urbana, IL, 61822, USA
[2] Carl R. Woese Institute for Genomic Biology, University of Illinois at Urbana-Champaign, Urbana, IL, 61822, USA
[*] corresponding author: vddonk@illinois.edu; 217 244 5360

Table of Contents

General **1**
Primers and site-directed mutagenesis **2**
Heterologous expression and purification **2**
N-ethylmaleimide (NEM) Alkylation Assays **3**
LC-MS and LC-MS/MS Sample Preparation **3**
Multiple Sequence Alignments from Glutamyl Lyase SSN **4**
MALDI-TOF MS, hrMS, and MS/MS characterization of mCoiA1 mutants **11**
GC-MS Analysis **15**
LC-MS Analysis **15**
Bioinformatic **19**
Analysis of OlvA and CoiA1 precursor peptides **19**
Genome Neighborhood Analysis of Glutamyl Lyase SSN **28**
References **29**

General

MALDI-TOF MS, LC-MS, LC-MS/MS, and GC-MS experiments were carried out as reported previously.[1] DNA sequences corresponding to peptide and proteins in this study can also be found in a previous report.[1]

Primers and site-directed mutagenesis

Site-directed mutagenesis was performed using NEBaseChanger using Q5 polymerase according to NEB protocol on a 50 µL scale. All PCR products were directly used in the next step by incubating 5 µL of the PCR product with 1 µL T4 DNA ligase reaction buffer (10X), 1 µL T4 DNA ligase (400,000 U/mL), 1 µL PNK (10,000 U/mL), and 1 µL DpnI (20,000 U/mL). The mixture was incubated at 37 ºC for 3 h. After 3 h, chemically competent *E. coli* DH10B cells were transformed with the mixture and plated onto solid lysogeny broth (LB) agar and incubated overnight at 37 ºC. A single colony was then picked and grown overnight at 37 °C in 5 mL of LB media containing the appropriate antibiotic(s). Plasmid was isolated using Qiagen Plasmid Kits and submitted for DNA sequencing. Primers used for mutagenesis experiments are listed in the table below.

Table S1. Primers used in this study.

Primer	Sequence (5' -> 3')
CoiB H994A FP	CTCATTATTGgccGCCCACCACATTC
CoiB H994A RP	TCTAAGACAAGATCTAAGTCG
$CoiS_{A(ED)}$ R51A FP	TCCTTGGTGGgccCTACGCTATC
$CoiS_{A(ED)}$ R51A RP	TGCTTCCGTAGAAAACTC
$CoiS_{A(ED)}$ R53A FP	GTGGCGACTAgccTATCGTCCGG
$CoiS_{A(ED)}$ R53A RP	CAAGGATGCTTCCGTAGAAAAC
$CoiS_{A(ED)}$ E89A FP	ATATGAGCCGgccGAAACGGCGTTT
$CoiS_{A(ED)}$ E89A RP	ATTCCACGGGTCCAACTC
CoiA1 T43S FP	GATTAACTTGagcGATGATGGATGTGGCTCCACTTGCAGC
CoiA1 T43S RP	AGGGTCGCAGAGCCGTCG
CoiA1 T50S FP	ATGTGGCTCCagcTGCAGCAGCC
CoiA1 T50S RP	CCATCATCCGTCAAGTTAATC
CoiA1 T57S FP	CCCGTGCGCGagcAATGTGGCGTAA
CoiA1 T57S RP	CTGCTGCAAGTGGAGCCA

Heterologous expression and purification

Heterologous production and purification of mCoiA1 and mCoiA1 mutants was performed as previously described for mCoiA1 on a 1 L scale for analysis.[1] For large scale isolation of products, co-expression experiments were carried out on an 8 L scale. Final yields varied from 0.3 – 1 mg/L. The following plasmids were used for co-expression experiments: pRSFDuet-1 His_6-SUMO-CoiA1_CoiB and pETDuet-1 CoiC_$CoiS_{A(ED)}$ with either CoiA1 or $CoiS_{A(ED)}$ mutants. His_6-SUMO tag was removed from the peptides using TEV protease (1:20 peptide: protease) in 20 mM Tris, 500 mM KCl, pH 7.5 buffer. The sample was incubated overnight to allow for full removal of His_6-SUMO tag. After overnight incubation at room temperature, trifluoroacetic acid was added to a final concentration of 2%, the sample was centrifugated, and the supernatant isolated. The desired peptide was purified by preparative HPLC.

Table S2. Observed and calculated m/z ratios for species observed by MALDI-TOF MS in this study.

Figure	Species	Theoretical m/z	Observed m/z
Figure 4	CoiA1 – 3 H_2O	5773.6	5771.9
	CoiA1 – H_2O	5809.6	5810.0
	CoiA1	5827.6	5827.9
	CoiA1 – H_2O + Glu	5938.6	5938.9
	CoiA1 + Glu	5759.5	5956.2
	CoiA1 – H_2O + 2 Glu	6067.8	6068.0
	CoiA1 + 2 Glu	6085.7	6086.1
Figure 6	CoiA1 – 3 H_2O	5773.6	5776.0
	CoiA1 – 2 H_2O	5791.6	5793.0
	CoiA1 – H_2O	5809.6	5811.0
	CoiA1 – 2 H_2O + Glu	5920.6	5922.3
Figure S2	CoiA1 – 3 H_2O	5773.6	5772.5
	CoiA1	5827.6	5827.0
	CoiA1 + Glu	5956.6	5957.1
	CoiA1 + 2 Glu	6085.7	6086.5
Figure S3	CoiA1 – 3 H_2O	5773.6	5770.4
	CoiA1 – 3 H_2O + NEM	5898.7	5896.4
	CoiA1 – 3 H_2O + 2 NEM	6023.8	6021.1
Figure S4	CoiA1 T43S – 3 H_2O	5759.5	5758.9
	CoiA1 T50S – 3 H_2O	5759.5	5759.6
	CoiA1 T57S - H_2O	5795.6	5795.3
	CoiA1 T57S – 2 H_2O	5777.6	5778.0
	CoiA1 T57S – 3 H_2O	5759.5	5760.0

N-ethylmaleimide (NEM) Alkylation Assays

NEM alkylation assay was performed by incubating 100 µM peptide in a 100 µL solution containing 10 mM tris(2-carboxyethyl)phosphine (TCEP), 10 mM NEM, 50 mM Tris, and 500 mM NaCl at pH 7.5. The sample was incubated for 3 h at room temperature and then analyzed by MALDI-TOF MS.

LC-MS and LC-MS/MS Sample Preparation

mCoiA1 (or mCoiA1 mutant peptide) (100 µM) was incubated with 2 µM GluC in 50 mM Tris, pH 8 in a total reaction volume of 50 µL. After overnight incubation, the sample was subjected to LC-MS using a AdvanceBio Peptide Plus (2.7 µm particle size, 150 x 2.1 mm) column.[1]

Table S3. Observed and calculated m/z ratios for fragments of GluC-digested mCoiA1 (for annotated ions in **Figure 3B**).

Ion	Observed m/z	Calculated m/z	Error (ppm)
b_5-H_2O	444.1315	444.1361	10.357
b_6	533.1843	533.1838	0.938
b_7-H_2O	616.2170	616.2209	6.329
b_7	634.2274	634.2315	6.465
b_8	747.3184	747.3155	3.881
b_9-H_2O	842.3865	842.3890	2.968
b_9	860.3997	860.3996	0.116
b_{10}	974.4421	974.4425	0.410
b_{11}	1087.5200	1087.5266	6.069
$[M+2H]^{2+}$	1361.0593	1361.0573	1.469
b_{17}	1617.6646	1617.6697	3.153
y_{18}	1634.5871	1634.5800	4.344
y_{20}	1861.7096	1861.7150	2.901

Multiple Sequence Alignments of Groups of Glutamyl Lyases

Group 1 Alignments

```
NISB                           REKLPFNEWLYLKLYISINRQNEFLLSYLPDIQK--IVAN-----LGGNLFFLRYTDPK- 780
MIBB                           PTG-AGGPWLYLRLRVPRRNQDDFLRDQVP----VLVRAG--IEHGADRWFFIRYSDTAG 864
COIB                           GHLPGAASWLLVKLYGHVERQPEILADHLP----ALLGQW----DAPPTWWYIRYRDPR- 809
TR|A0A1N7KLA3|A0A1N7KLA3_9FLAO RSFAPGSEWLYLKMYCNSNVSDYLLANEVTPLLDHLIDEK-----MIKSAFFIRYTDPH- 783
TR|A0A316HG90|A0A316HG90_9SPHI RNFFPGSEWLFIKLYLSSNIANNIILR-LNELLDHS-GTV-----SIKQWFFIRYHDPD- 778
TR|A0A481XVF7|A0A481XVF7_9PSEU PAAARAANWVYFQYFAGQAASDEVVDRAAA-LAEELRGQG-----LVGDWFFLRYQDDG- 768
TR|A0A0N1JZV9|A0A0N1JZV9_9ACTN RHL-PGGQWLYAKLYVPEHHQPAVLARHLG----RLTGPALASIAGIDTWFFLRYADPA- 770
TR|A0A4Q8N062|A0A4Q8N062_9STAP GHPQLKRDWLSVHLFIEESYQNEFIIQYLLPFVSNLLADN-----QLENFFFIKYKDNK- 773
TR|A0A521FN28|A0A521FN28_9SPHI RSFLPGEEWLYIKLYAHYSLTDEILLNFILPVIRKYKKDN-----PEFKWFFIRYMDPG- 768
TR|A0A540PBM2|A0A540PBM2_9ACTN ARMPGASPWVYLKLYGNAAQVPELLTAHVP----LLLGEV-----APLGWWFIRYADPD- 771
                                       *:  :          .:                         ::::* *

NISB                           PHIRLRI--KCSDLF-LAYGSILEILKRSRKNRIMSTFDISIYDQEVERYGGFDTLELSE 837
MIBB                           QHLRVRFRGEREKLWAGLLPEIGARLVEWQRQGLLAGHELGQYDPEYERYGGDALAEFTE 924
COIB                           WHLRLRIAVPSEQDFALTAQRVSAWANGLRRAGLLTDMQFATSYPETGRWGPGPLMGLAE 869
TR|A0A1N7KLA3|A0A1N7KLA3_9FLAO YHLRLRLNLCDKKLYAEVLQKLYDSLDPYFQSEMIWNLQIDSYQRELERYDP-EYIEDTE 842
TR|A0A316HG90|A0A316HG90_9SPHI NHIRVRINLLKEDDFQDVYLAIREVLSPFLISGQVHVVQLDTYVRELERYGN-QIEQA-E 836
TR|A0A481XVF7|A0A481XVF7_9PSEU YHVRVRMRATSPAARPSVLTAMDAFGRRLRATGLATRAVLRDYVPEIARYGGEAGLPLAE 828
TR|A0A0N1JZV9|A0A0N1JZV9_9ACTN PHLRLRFHGKPEALWSELLPALRTWTEELTDAGLLSRLTLDTYEPEVHRYGGPAALAHAE 830
TR|A0A4Q8N062|A0A4Q8N062_9STAP HFIKLRLLNKSRDLS-LLYRKFLEHKQIWLKESELSHYAIVDYQPEIHRYGGIQSIENIE 832
TR|A0A521FN28|A0A521FN28_9SPHI HHLRLRFYTGQRPSY-ELLSDLIHKLQPWSKEGKVSDVILDSYQRELEKYSV-SLINEVE 826
TR|A0A540PBM2|A0A540PBM2_9ACTN SHVRLRLRLPSPEGFGDAARCVAVWAAELRNEGLLQRVRWDTDEPETGRYGTGAVLEAAE 831
                                .:::*:               .                       *  ::.        *
```

```
NISB                            DFELQKVYSIIDSIIHVHNNRLIGIERDKEKLIYYTL-QRLFVSEEYMK----------- 993
MIBB                            TDSPSTQLRLVGSLLHMTCNRLIGGSAERE-RSVLGLARGAVQDNLNRRRHLA------- 1115
COIB                            --GGIDPDVVLDSLLHAHHIRAAGIDKDDE-RMCVRLAHAAAMAWTHRGDHHESA----- 1032
TR|A0A1N7KLA3|A0A1N7KLA3_9FLAO  KLC----KEKLSSYIHMSINRWFPSEQRALELMTYSFATKYYSRILSQTR---------- 993
TR|A0A316HG90|A0A316HG90_9SPHI  DWN----DKFLSSVIHMAVNRYFSTRQRLHECILYGIMLKYLKSEKARNKL--------- 993
TR|A0A481XVF7|A0A481XVF7_9PSEU  --PERRAWSVFGSALHLHCNRMFAFDAVRLEYLAHELAIRAVRRRKALDGNGTSGERGAA 992
TR|A0A0N1JZV9|A0A0N1JZV9_9ACTN  EGGSAGLPAVAQSLAHMHCNRFLGLDRDAE-MLAHATAREALALRLNRMRHGR------- 992
TR|A0A4Q8N062|A0A4Q8N062_9STAP  QALSTPRTHIIGSLIHMRCNRIFGVNRDKEKFVLSIF-NEIEKTKKYWCGETING----- 993
TR|A0A521FN28|A0A521FN28_9SPHI  DWSREARYNLLINLVHMHVNRIFENTPREYEYLIYHFMKKHQSFLNYTTTDGVS------ 993
TR|A0A540PBM2|A0A540PBM2_9ACTN  ---GTDPAAVLPSLLHMHHNRTAGIAPDAE-ATCRRLARTAALSWTVRTEGAMR------ 993
                                            .  *    *
```

Group 2 Alignments

```
NISB                            ERREKLPFNEWLYLKLYISINR--QNEFLLSYLPDIQKIV-ANLGGNLFFLRYTD-PKPH 782
MIBB                            REPTG-AGGPWLYL--RLRVPRRNQDDFLRDQVPVLVRAGIEHGADRWFFIRYSDTAGQH 866
COISA                           -----MAADEWPQRLIRFTDWSRAEATAVEHLLPVLIAH--ESELAQWSFLRKFPWW--- 50
TR|A0A2S4Y379|A0A2S4Y379_9ACTN  RAALDVRSVGWHQLNIQFTDYSTAERTFRSCLVPALRN----GSVGSWWFVRKYPCW--- 100
TR|A0A365ZRN4|A0A365ZRN4_9ACTN  LAALDTQPAGWHQVNIEFADYPTAEHAFRTSLLPSLRT----GPVGAWWFVRKYPCW--- 100
TR|A0A1C4Y6S9|A0A1C4Y6S9_9ACTN  EPPGVAPASRWRQINLTLSDWQSAEDFAATRLAPELAAAEDHRTITAFWFIRKSETW--- 66
TR|A0A1C5DGR4|A0A1C5DGR4_9ACTN  -----MPDDEWPQRVIEFTDWPDAERVAVAHLRPILDS----GGTRQWSFIRKAPTW--- 48
TR|A0A3R7EXA6|A0A3R7EXA6_9ACTN  RAALGPHTSGWHQVNIEFADYPTAERSFRAHLLPALST----GPIGEWWFVRKHPCW--- 100
TR|A0A1Q5BUQ5|A0A1Q5BUQ5_9ACTN  -----MPPDRWQQHNITFADRESAQRAVSERIAPVLLAAEQDGQLAGWWFMNKQP-W--- 51
TR|A0A239LUX7|A0A239LUX7_9ACTN  -----MSPTLWHQHNVVFPHRRTAQQVIAERIGPTLELAQEADQLTGWWFMNKQP-W--- 51
TR|A0A429NGF1|A0A429NGF1_9ACTN  RAALDTRTTGWYQTYITFADYRTAEDAFRTHLLPALRS----EPVGAWWFVRKHPCW--- 100
TR|A0A540PMA9|A0A540PMA9_9ACTN  RSALDPQLSGWHQVNIKFTDYPTAERTFRAYLLPALRT----GPIGAWWFVRKYPCW--- 100
                                          *      :      :        * :             *:.

NISB                            IRLRIKCSDLFLAYGSI---LEILKRSRKN-RIMSTFDISIYDQEVERYGGFDTLELSEA 838
MIBB                            LRVRFRGEREKLWAGLLPEIGARLVEWQRQ-GLLAGHELGQYDPEYERYGGDALAEFTET 925
COISA                           -RLRYRAAGPDTAK----ALDAALDELVDA-GVLASWTRGIYEPEETAFGGPAAMKIGHT 104
TR|A0A2S4Y379|A0A2S4Y379_9ACTN  -RLRLCPSPQAPQEDAIAPITEALEGSVAR-GAVAAWRRFPYEPETVAFGGPAGMKIAHE 158
TR|A0A365ZRN4|A0A365ZRN4_9ACTN  -RLRVQPDTDARSEDTVSHIAEALDHAASR-GVVKRWWPTLYEPETFAFGGPYGMTLAHT 158
TR|A0A1C4Y6S9|A0A1C4Y6S9_9ACTN  -RLRLLPGDRLA------QVYALLASITDD-DRIRGVTEPVYRPEAYAFGGDQAMTIAHT 118
TR|A0A1C5DGR4|A0A1C5DGR4_9ACTN  -RLRYRPDP-ADPT----YLDQALDTLVAD-GTVREWVPGIYEPETAAFGGPDGMELAHE 101
TR|A0A3R7EXA6|A0A3R7EXA6_9ACTN  -RLRIHPGPDTRAEDALARIAEALDSPLAR-GVIRRWRPSLYEPESTAFGGPEGTRLTHS 158
TR|A0A1Q5BUQ5|A0A1Q5BUQ5_9ACTN  -RLRYRADEPSP------AVEAVLSDLVQDGGPVVSWLPCVYEPETEAFGGTDAMEVAHE 104
TR|A0A239LUX7|A0A239LUX7_9ACTN  -PLRYRAAQPSE------LIETLLTSLVHDG-TALAWHPAIYEPETDAFGGPHAMATAHQ 103
TR|A0A429NGF1|A0A429NGF1_9ACTN  -RLRVHPPPHTPVEDTVAHVTKALDTAVSR-RAVKEWSPSLYEPETVAFGGTEGMTITHA 158
TR|A0A540PMA9|A0A540PMA9_9ACTN  -RLRVQPGPGTRMEDAVAHLTEVLDRALSW-GVAKRWWPFLYEPETIAFGGPYGMTLTHT 158
                                  :*                   *                 *  *   :**       .

SP|P20103|NISB                  YDKN------FKELKHAIKNLFLKMIAQDFELQKVYSIIDSIIHVHNNRLIGIERDKEKL 977
TR|E2IHB7|MIBB                  PTGGWPLLRADEDGCQVLAAL----ESRDEAVRR-------------F-G-------- 1054
COISA                           PLAEWIAT--FEWFGQQLADL----NRQGRLERGLRAVIAHHGIFHFNRL-GLPAQDQRT 252
TR|A0A2S4Y379|A0A2S4Y379_9ACTN  PVRDWFQG--LEQGGLALADA----WNNGRLALGMRGVLARHVLFHWNRM-GFTLRQQSI 306
TR|A0A365ZRN4|A0A365ZRN4_9ACTN  PLQTWFTE--MRSTGRALGNA----APAGSLHLGLRSILARHILFHWNRM-GFTTHQQAI 306
TR|A0A1C4Y6S9|A0A1C4Y6S9_9ACTN  ITPQWPRA--QEQAGADLGFL----DRHGALTRDLREVLTHHLLFLFNRL-GISAADTWL 257
TR|A0A1C5DGR4|A0A1C5DGR4_9ACTN  YLTDWVTT--FEWAGQQLAHL----NRRGRLERGLRAVLAHHLIFHWNRL-GLPREDQSA 250
TR|A0A3R7EXA6|A0A3R7EXA6_9ACTN  PLARWTTG--MQRGGRALAEA----AHVGSLQLGLRSVLARHILFHWNRM-GFTAAQQAV 306
TR|A0A1Q5BUQ5|A0A1Q5BUQ5_9ACTN  GHGGWVAA--FERAGAELAAL----AGRGALTRGLRAVIAHHTIFHSNRA-GLLRGDQSA 249
TR|A0A239LUX7|A0A239LUX7_9ACTN  GHSTWVDA--FEEAGATLADL----GRCGQLTRGLRAVIAHHVIFHANRA-GLPLDDQSA 247
TR|A0A429NGF1|A0A429NGF1_9ACTN  PLRPWATG--MQHGARALATA----ARDGRLGLGLRGILARHVLFHWNRM-GFTPRQQAI 306
TR|A0A540PMA9|A0A540PMA9_9ACTN  PLGYWVTG--VERSGQALVDA----ARTGNLHLGLRGILARHILFHWNRM-GFTTRQQAI 306
                                           .     :          .                      *
```

Group 10 Alignments

```
NISB                           RYTDP-KPHIRLRIKCSDLFLAYGSILEIL-----KRSRKNRIMSTFDISIYDQEVERYG 828
MIBB                           RYSDTAGQHLRVRFRGEREKLWAGLLPEIG--ARLVEWQRQGLLAGHELGQYDPEYERYG 915
SPTBB                          HK----PPGLRVRFLAADPSRAPELRADLL--RRLTPERTGEAGTPPVTGVYEPETYLFG 144
TR|A0A022MI87|A0A022MI87_9ACTN HK----PPGLRVRFRAAHPSRAGELRAALT--RRLDPATEDAPWTRPVAEVYEPETYLFG 148
TR|A0A1T3NRC9|A0A1T3NRC9_9ACTN HK----PPGVRIRFAVHGDAATAERLREIV-SARADDWLREHVVRHREPGGYEPEAHLFG 164
TR|A0A1E7K2X1|A0A1E7K2X1_9ACTN HQ----DPAVRVRFQAFGPQDAAELRTALL--HRFARC--QGARTRPVPVVYEHEHYLFG 127
TR|A0A1H4QN90|A0A1H4QN90_9ACTN DK----EPGLRIRFRFCEVDIERQQRAD-L-ESRIVEWQSRGIINGWSKSFYEPEQYLFG 158
TR|A0A5B8IEG5|A0A5B8IEG5_9ACTN HK----PPGLRIRFQAGRLGDVPGLRAELL--DRTA------EFGDVVATVYEPEAYLFG 98
TR|A0A6G4U964|A0A6G4U964_9ACTN RK----PPGLRVRFRCAEGRADA-VDER-V-RTALDEWRRDGLVAAWSPGAYEPEEFLFG 115
TR|A0A4Y3R5G8|A0A4Y3R5G8_STRCI HK----APGLRVRFQAPGPCPQ-ALHRALL--RRFRGT--PGLRGEPVRAVYEPEHYLFG 157
TR|A0A7W7SNY7|A0A7W7SNY7_9ACTN HK----PPGLRIRFETATGQHQP-LHDA-L-HTRLDRLRT--HLDQLTPGIYEPEQLLFG 142
TR|A0A6G2QXF1|A0A6G2QXF1_9ACTN HK----PPGVRLRLRACSGRERD---LALLTTRQARSLSRAGVVSGYAPGVYEAESCLFG 183
                                        :*:*:                                     *: *   :*

NISB                           YSIIDSIIHVHNNRLIGIERDKEKLIYYTLQRLFVSEEYMK------------- 993
MIBB                           LRLVGSLLHMTCNRLIGGSAERERSVLGLARGA-VQDNLNRRRHLA-------- 1115
SPTBB                          -ATVGPRRAAAHHVIF----HWNRGALSTARQCLLTEALAADGREEAD------ 320
TR|A0A022MI87|A0A022MI87_9ACTN -ATVGPRRAAAHHVIF----HWNRGRLSTARQCLLTEALAADGHDGTD------ 323
TR|A0A1T3NRC9|A0A1T3NRC9_9ACTN -AEVGPRQALAYYTVF----HWNRGRLALPTQCLLADALAGRTRS--------- 333
TR|A0A1E7K2X1|A0A1E7K2X1_9ACTN -ATVGPRTAAACFTAS----HWNRGRLSPARQRLLTHVLAAEEGAPHAPPPPSR 313
TR|A0A1H4QN90|A0A1H4QN90_9ACTN -ARIGPREAAAFYIAY----HWNRARIPFVWQCAIAEALAK------------- 323
TR|A0A5B8IEG5|A0A5B8IEG5_9ACTN -AVRGPRRAAAYAVIF----HWNRAALPATRQCLLTEALASDGVV--------- 271
TR|A0A6G4U964|A0A6G4U964_9ACTN -PAVGPREAAAFVIVF----HWNRAGLSAVRQALITSALTARPRWAER------ 287
TR|A0A4Y3R5G8|A0A4Y3R5G8_STRCI PVEVGPRRAAAYFTVF----HWNRGLVSLARQNLITEALAGEGSRHAARA---- 346
TR|A0A7W7SNY7|A0A7W7SNY7_9ACTN -ALIGEREAAAFVTIF----HWNRGRLSALTQALVAAALADRTSRP-------- 312
TR|A0A6G2QXF1|A0A6G2QXF1_9ACTN -ALIGPREAASLAIVF----HWNRAGIAPMRQALITEALADRYAWVPT------ 358
                                   .               . ::          :
```

Group 14 Alignments

```
NISB                           PFNEWLYLKLYISINRQNEFLLSYLPDIQKIVA--NLGGNLFFLRYTD-PKPHIRLRIKC 789
MIBB                           AGGPWLYLRLRVPRRNQDDFLRDQVPVLVRA-GIEHGADRWFFIRYSDTAGQHLRVRFRG 873
TR|A0A365XRZ7|A0A365XRZ7_9BACT MQRSWLSVHLFYAGDLNHLLHLLVAPVVAQI------EAPFFFIRYWE-GGPHIRLRLYV 53
TR|A0A1T4MF28|A0A1T4MF28_9BACT MESNWLSVHLFHAADLNRLLQLLVGPVVQQT------GCPYFFIRYRE-GGQHIRLRLHV 53
TR|A0A1G9SCU0|A0A1G9SCU0_9SPHI MKSKWFAIHIYYPGNLDLMLQQLIHPFINRFFKNES-DGAYFFIRYWE-NGSHIRLRMKV 58
TR|A0A4V2JGH2|A0A4V2JGH2_9SPHI QQDKWLSVYIFHQGDANELLKQLVHPFIQQW------NAPWFFIRYWE-GGDHIRLRLKA 56
TR|A0A366L1D9|A0A366L1D9_9SPHI QEEKWLSLYIFYQENADEILKQVVHPFIRQW------HNPWFFIRYWE-GGDHIRLRLKT 74
TR|A0A433WL85|A0A433WL85_9BACT TGTTWLSAHIYSRGPLDRLINLLAAPFTATVAACLHPVSPFFFIRYGE-GGPHIRLRLHV 77
TR|A0A3N7F4I3|A0A3N7F4I3_9SPHI METKWFAVYLFYPGDLDLMLNQLVQPFIHDFFKEGS-AETYFFIRYRE-NGSHIRLRMKV 58
TR|A0A7Y0NQA4|A0A7Y0NQA4_9SPHI HKEKWFSLYIFYHENADRLLIELIHPFIWQW------RRPWFFIRYWE-GGNHIRLRLKA 56
TR|A0A7W8YVQ5|A0A7W8YVQ5_9SPHI KAIQWFAAYLFYAGDLDLMLRELVTPFVCEFFPHDNTEASWFFIRYWE-NGSHIRLRMNA 62
TR|A0A6B9ZP14|A0A6B9ZP14_9BACT MQTVWLSAHLFYAGDLHVLLRELVIPFLKDR------GCPAFFIRYGE-GGLHIRLRCQL 53
                                   *:   :      .  :     *               **:** :    *:*:*
```

```
NISB                           SDL----------------FLAYGSILEI-LKRSRKNRIMSTFDISIYDQEVERYGGFDT 832
MIBB                           EREK---LWAGLLPEIGARLVEWQRQ-----------GLLAGHELGQYDPEYERYGGDAL 919
TR|A0A365XRZ7|A0A365XRZ7_9BACT DETAEIQVKEYLEAASDRYFLQYPSQRRE-LAYQTPLLPNDSLQYITYVPEISRYGDERS 112
TR|A0A1T4MF28|A0A1T4MF28_9BACT APGSLTEIRRLLQDAAQAYFTAYPSCREK-AFPTQQLQPNDTLQYIPYVRETSRYGNEQT 112
TR|A0A1G9SCU0|A0A1G9SCU0_9SPHI DPEKQEMLSAEISNSVNAFFSQYPGPMLHTGPETASESPGHELTYTSYEPEINRYGNQQS 118
TR|A0A4V2JGH2|A0A4V2JGH2_9SPHI AVSQHDLIVTSLSSEKSI---------------------IRSVQIAKYEPEIDRYGNIES 95
TR|A0A366L1D9|A0A366L1D9_9SPHI PKIEHDEIIKRLKLGNTG---------------------IKSLQIAQYEPEIDRYGNRES 113
TR|A0A433WL85|A0A433WL85_9BACT NTSHTETVRHMLKEAAGNYFSEYPSVPVS-G---GEMPLHDTLAFMPYQQETARYGNQHT 133
TR|A0A3N7F4I3|A0A3N7F4I3_9SPHI LPETQAMLELEINQRAAGFFVRYPELTLPQDLAATTAPPGHKVVYSSYEPEIKRYGNLQS 118
TR|A0A7Y0NQA4|A0A7Y0NQA4_9SPHI SENEHNAIVKALSPGNAA---------------------IKTIQTARYEPEIDRYGNYES 95
TR|A0A7W8YVQ5|A0A7W8YVQ5_9SPHI ELSLQNVLIKTLDKRANEFFLQYPA--------------DHALQFPVYEPEITRYGNHQS 108
TR|A0A6B9ZP14|A0A6B9ZP14_9BACT KVSDVTGVQVALL-------------------AMENIFEGLTVRFTDYIPEINRYGDARS 94
                                                                              *  *  ***.

NISB                           --Y-----------DKNFKELKHAIKNLFLKMIAQDFELQKVYSIIDSIIHVHNNRLIGI 970
MIBB                           DEDGCQVLAALESRDEAVRRFGTAYREAFRPT---D-SPSTQLRLVGSLLHMTCNRLIGG 1088
TR|A0A365XRZ7|A0A365XRZ7_9BACT GRT-----------TDVLQTFADGNRIIFQQYRLLGFSNRQLGDITGSFIHMGHNRLGVS 269
TR|A0A1T4MF28|A0A1T4MF28_9BACT GNV-----------PETLQAFAAGNSHVFAQYKQLGIQQEQLGVIARSFLHMGHNRLGVA 263
TR|A0A1G9SCU0|A0A1G9SCU0_9SPHI DTA-----------PEKVSRFLQENLKIMKNYSAAGFEEAKLNEIVNSMMHMNNNRLGIS 274
TR|A0A4V2JGH2|A0A4V2JGH2_9SPHI REV-----------DDDLDAYLSETISVMSLYKCTVFEEHKLFQVISSFKHMNNNRLGIS 251
TR|A0A366L1D9|A0A366L1D9_9SPHI GIA-----------EDNLMDYLSNTNQVIRLYQNAGFKKDILFQVISSFMHMNNNRLGIS 269
TR|A0A433WL85|A0A433WL85_9BACT GKA-----------PPALQSFTYRNSRLLQRYYHAGLAAGQLEEAFCSMMHMSHNRLGLS 287
TR|A0A3N7F4I3|A0A3N7F4I3_9SPHI DAA-----------SDKISRYTHENKSIMKNYLSAGFEETKLTEIVTSMMHMNNNRLGIS 274
TR|A0A7Y0NQA4|A0A7Y0NQA4_9SPHI DIV-----------DDDLMEYLSKTTKVICSYQKIDFEKNKMFQVISSFMHMNNNRLGIS 251
TR|A0A7W8YVQ5|A0A7W8YVQ5_9SPHI GTA-----------NQEVQDLFKANRLILQNYQQADFTEIKRIEIMTSLMHMNNNRLGIS 264
TR|A0A6B9ZP14|A0A6B9ZP14_9BACT ENA-----------DSSLQVFAEENKHVFRQYRGLGFDTSKMRDIVGSCMHMGHNRMGVS 252
                                                .          :                  *  *:  **:
```

Group 19 Alignments

```
NISB                           PFNEWLYLKLYISINRQNEFLLSYLPDIQKIVAN-LGGNLFFLRYTD-PKPHIRLRIKCS 790
MIBB                           AGGPWLYLRLRVPRRNQDDFLRDQVPVLVRAGIEHGADRWFFIRYSDTAGQHLRVRFRGE 874
TR|A0A1G9VKH3|A0A1G9VKH3_9ACTN GTREWRVVHIHVPHSLHTPFLCDVVDPLLRAEGL--RDDFFFLRYWQ-GGPHLRLRMRCG 68
TR|A0A5R9E8E6|A0A5R9E8E6_9ACTN GTREWRVVHIHVPHSLHTPFLCDVVDPLLRAEGL--RDDFFFLRYWQ-GGPHLRLRMLCG 97
TR|A0A399HM37|A0A399HM37_9ACTN RVAEWRVVHIHVPHSAHTPFLCDVVGPLLRQEGL--QDHFFFLRYWQ-GGPHLRLRMLCG 68
TR|A0A8A3HC15|A0A8A3HC15_9ACTN GTAQWRPVHIHIPPSLHTSFLCDVVGPLLEDEGL--RDHFFFLRYWQ-GGPHVRLRMLCG 68
TR|A0A3P1V584|A0A3P1V584_9ACTO PKRSWHCWHAYLPHSRANDFLTALR-AQQRSRIE--AHDFFFLRYWQ-GGPHVRVRFRLE 59
TR|A0A387HBE5|A0A387HBE5_9ACTN RADGWRALHVHLPHSLQTAYLRDVIRPVVRAGAQ--GERFFFLRYWQ-GGPHIRLRIGG- 63
TR|A0A2N8P741|A0A2N8P741_STRNR -------------MSLQPGFLRDVLHPVMREAGM--RNRFWYLRYWQ-GGPHIRLRLHE- 43
TR|A0A0G3AKI1|A0A0G3AKI1_9ACTN -------------MSLQPTFLRDVIRPVVRDAGM--RERFWYLRYWQ-GGPHIRLRLHR- 43
TR|A0A085B5B8|A0A085B5B8_CUTAC DPGGWRALHIHLPPSVQSEYLADVILPSMSALMP--EHDYFFLRYWQ-GGSHLRLRLRST 66
TR|J1ZVD5|J1ZVD5_9ACTN         -------------MSLQPGFLRDVIHPVVREAGV--RNRFWYLRYWQ-GGPHIRFRLHE- 43
                                                  :*                   :::** :    *:*.*:

NISB                           ISIYDQEVERYGGFDTLELSEAIFCADSKIIPNLLTLIKDTNNDWKVDDVSILVNYL--- 873
MIBB                           LGQYDPEYERYGGDALAEFTETAFQHDSAAAISLLRLTRRAGFRYTLDEVTAISAAALAD 963
TR|A0A1G9VKH3|A0A1G9VKH3_9ACTN RVPYEPEYRKYGGPEGVEIAETVFRKSSAAVLDLLGRQPRAWVEER------------RA 175
TR|A0A5R9E8E6|A0A5R9E8E6_9ACTN RVPYEPEHRKYGGPEGVEIAETVFRKSSAAVLDLLGRQPRAWVEER------------RA 204
TR|A0A399HM37|A0A399HM37_9ACTN RAPYAPEYRKYGGRQGLDIAEAVFRRTSAAVLDMLDRRPPGRPGAA------------RA 175
TR|A0A8A3HC15|A0A8A3HC15_9ACTN PVTYEPEFRKYGGTEGVAIAETVFRKSSVAVLDLLARQPAAA-GAG------------RA 174
TR|A0A3P1V584|A0A3P1V584_9ACTO PHPYAPEYAKYGGAGGVAIAEDLFCSGTCAVLDLLAAAEGG----P------------FD 157
TR|A0A387HBE5|A0A387HBE5_9ACTN PMAYAPEYAKYGGAEGVRIAEELFSRTSVAVLDLAAARPTP--E-P------------KA 164
TR|A0A2N8P741|A0A2N8P741_STRNR AAVYSPEYAKYGGTTGLRIAEELFRSTSTAVLDLLAGLTDR--QLA------------AS 145
TR|A0A0G3AKI1|A0A0G3AKI1_9ACTN PSPYSPEYAKYGGTTGLRIAEELFRSTSAAVLDLLADRSDR--ELQ------------AS 145
TR|A0A085B5B8|A0A085B5B8_CUTAC ERRYQPEYRKYGGSVGVSIAEDVFCSTSQSVLRILNERPIS----H------------RQ 170
TR|J1ZVD5|J1ZVD5_9ACTN         AARYSPEYAKYGGTTGLRIAEELFRSTSTAVLDLLAGLTDR--QLT------------AS 145
                                  *  *  :***     ::*  *   :     :

NISB                           --IAQDFELQKVYSIIDSIIHVHNNRLIGIERDKEKLIYYTLQRLFVSEEYMK------- 993
MIBB                           PSTQ--------LRLVGSLLHMTCNRLIGGSAERERSVLGLARGAVQDNLNRR---RHLA 1115
TR|A0A1G9VKH3|A0A1G9VKH3_9ACTN DARDLRLDGTPYSGCLSNYVHTTNNRLGLVPA-AEGLVAYLVRRALAELPE--------- 320
TR|A0A5R9E8E6|A0A5R9E8E6_9ACTN DARDLRLDGTPYSGCLSNYVHTTNNRLGLVPA-AEGLVAYLVRRALAELPE--------- 349
TR|A0A399HM37|A0A399HM37_9ACTN DVRELLLDATPYPGCLSNYVHTTNNRLGLLPA-AEGLVAYLVRKGLEESAG--------- 320
TR|A0A8A3HC15|A0A8A3HC15_9ACTN DVHELRLDGTPYLSCVSNYVHTTNNRLGLIPA-GEGLVAYLVRRGLEGLVG--------- 319
TR|A0A3P1V584|A0A3P1V584_9ACTO TRLDTVLDGAAFGSVLSNYIHTNNNRLGLRPD-QEIVVALLLARALEAQPEPPAGTRS-- 304
TR|A0A387HBE5|A0A387HBE5_9ACTN DLGDLVLGGTPYTRCLSNYLHTTNNRLGIIPA-GEAFVAHVMNRALSEIGTRTAI----- 311
TR|A0A2N8P741|A0A2N8P741_STRNR DVAELVLEETPYAHCLANYIHTTNNRLGILPV-AEAFLAYVIRRSLAEMATA-------- 290
TR|A0A0G3AKI1|A0A0G3AKI1_9ACTN DFGELALEGTPYAGCLANYIHTTNNRLGILPA-AEAFLAYVIRSSLTELRVPHKA----- 293
TR|A0A085B5B8|A0A085B5B8_CUTAC ARNTSVKIDPMFLSCLANYIHTTNNRLGLPPA-LESTAAYLVQLGFRSVAVREEKVR--- 314
TR|J1ZVD5|J1ZVD5_9ACTN         DLAELVLDETPYAHCVANYIHTTNNRLGILPA-AEAFLAYVIQRSLAELRGTTPDPNGIR 298
                                              : . :*   ***       *          .
```

Group 23 Alignments

```
NISB                           PFNEWLYLKLYISINRQNEFLLSYLPDIQKIVANL--GGNLFFLRYTDP-KPHIRLRIKC 789
MIBB                           AGGPWLYLRLRVPRRNQDDFLRDQVPVLVRAGI-EHGADRWFFIRYSDTAGQHLRVRFRG 873
TR|A0A1A6CDF5|A0A1A6CDF5_ELIMR MKKNWETYYLYYEDHADRVLKEIVHPVIEDIQYKLKKTVKFFFIRYFEN-GYHIRLRVLL 59
TR|A0A1T3FP13|A0A1T3FP13_9FLAO MKKNWETYYLYHEGNADQVLKEIVHPAIDEVQYKLKKTVKFFFIRYFEN-GYHIRLRLLL 75
TR|A0A7Y0AMJ2|A0A7Y0AMJ2_9FLAO MEERWHSYYIYHTGDADLLLKEVVHPSVHDIQEKLEKEVKFFYIRYFEN-GYHIRLRILL 59
TR|A0A086AY63|A0A086AY63_9FLAO MQKYWKTYYIYHTRNADDLLKEIIHPSLLDINEKLEKEVQFFFIRYFEN-GYHIRLRLLL 59
TR|A0A1M5GCP4|A0A1M5GCP4_9FLAO MKKIWNTWYLYYNGDADSLLKEIVHPSLENIEEKLKKEVKFFFIRYFEN-GYHIRLRLLL 59
TR|A0A220S8K3|A0A220S8K3_9FLAO MQKSWNTYYIYYGGSADQILKEIVHPSIEDVQQSMEKELKFFFIRYFEN-GYHIRLRLFL 59
TR|A0A2G6UK85|A0A2G6UK85_9FLAO MEKLWNTYYIYHPDDADRLLKEVVDPAVQNLQEVLTEEVKFFFIRYFEN-GYHIRLRLLL 59
TR|A0A4Q1EPL1|A0A4Q1EPL1_9FLAO DEKNWDTYYLYHKGNADEILKEIVHPAVENIQYSLKKTVKFFFIRYFEN-GYHIRLRLLL 60
TR|A0A0J7IFG7|A0A0J7IFG7_9FLAO MQKLWNTYYLYYKGDADHLLKEVVHTSLQNIQEQLGKEVRFFFIRYFEN-GYHVRLRLLL 60
TR|A0A135WIL0|A0A135WIL0_9FLAO MEKIWKTYYLFHENQADPVLTGVVHPLIQNIEKILEREIKFFFIRYFED-GYHIRLRLLL 59
                                   *    :      :  :       :           . *::** :    *:*:*.

NISB                           SDLFL-AYGSILE--I--LKRSRKNRIMSTFDISIYDQEVERYGGFDTLELSEAIFCADS 844
MIBB                           --EREKLWAGLLPEIGARLVEWQRQGLLAGHELGQYDPEYERYGGDALAEFTETAFQHDS 931
TR|A0A1A6CDF5|A0A1A6CDF5_ELIMR FPEESPLFRSILTYYI--SDS----DIKIILKEAQYIPETERYGNSDTIVYAENQFYASS 113
TR|A0A1T3FP13|A0A1T3FP13_9FLAO SPEESPLFRSLLTHHI--SDS----GIKIILKEAQYIPETERYGNSDTIVYAENQFYASS 129
TR|A0A7Y0AMJ2|A0A7Y0AMJ2_9FLAO SGEESVAFLSILKHFI--AAYEDFNGIHLISKEAQYIPETERYGNADTIAHAESQFCASS 117
TR|A0A086AY63|A0A086AY63_9FLAO NVEESTLFLTILMDHI--SDYEHLNECHLELKQAEYNPETERYGNANTITYAEDQFWASS 117
TR|A0A1M5GCP4|A0A1M5GCP4_9FLAO SDEESSVFISVLKDQI--SVYEKHHGVNLILKEALYIPETKRYGNADTILFAESQFDASS 117
TR|A0A220S8K3|A0A220S8K3_9FLAO SAKESILFKSMLANHV--SVYEHINKAAIVVRETEYIPEIGRYGNADTKIYAESYFYESS 117
TR|A0A2G6UK85|A0A2G6UK85_9FLAO SAEKAKVFLLILKDHI--SVYEQRNGVFLELKEAEYIPETERYGNENTIAHAENQFCASS 117
TR|A0A4Q1EPL1|A0A4Q1EPL1_9FLAO SSKESPLFISLLTHCI--SDYEFSHDTKIVLKKDQYIPETERYGNSETIMYAEDQFYASS 118
TR|A0A0J7IFG7|A0A0J7IFG7_9FLAO SADEVPLFFSVLKDQL--SNYERVHETQVVLKEAQYIPETDRYGNADTIAYAESQFYGSS 118
TR|A0A135WIL0|A0A135WIL0_9FLAO NIEESTIFLPLLKGQV--SIYEHTNKTSIILKEAQYIPETERYGNTATISYAENQFYASS 117
                                      :  :*                       *  *  ***.      :*  *  .*

NISB                           IKNLFLKMIAQDF---ELQKVYSIIDSIIHVHNNRLIGIERDKEKLIYYTLQRLFVSEEY 991
MIBB                           VRRFGTAYREAFRPTDSPSTQLRLVGSLLHMTCNRLIGGSAERERSVLGLARG-AV-QDN 1107
TR|A0A1A6CDF5|A0A1A6CDF5_ELIMR -REINKKYTDSHL---PAEAIDEALLSFIHMTNNNRIGIINSEESYLLFLVRQTITLIKDY 280
TR|A0A1T3FP13|A0A1T3FP13_9FLAO -REVNKKYTDSYL---PAEAIDEALLSFIHMTNNNRIGIINSEESYLLFLVRQTITLIKDY 296
TR|A0A7Y0AMJ2|A0A7Y0AMJ2_9FLAO -YRVWKEYTHSQL---PSDAVDEALLSFIHMTNNNRIGIVNAEESYLLFLLMKTLPLLKDY 284
TR|A0A086AY63|A0A086AY63_9FLAO -EKVWKDYTQAQL---SSEALDEALLSFIHMTNNNRLGIANAEESYLLFLILKTIPLIKDY 284
TR|A0A1M5GCP4|A0A1M5GCP4_9FLAO -HEVWKEYTQSQL---PENALNEAFLSFIHMTNNNRLGIINSEESYLLFLIKQTIPLIQNY 284
TR|A0A220S8K3|A0A220S8K3_9FLAO -GKIWSKYNQAQL---SSNALEEVLLSFIHMTNNNRLGIVNSEESYLLFLIMKTIPLIKDY 284
TR|A0A2G6UK85|A0A2G6UK85_9FLAO -HKIWQEYVQSKL---PLKALEEALLSFIHMTNNNRWGIINAEESYLLFLIMRTLPLIKDY 284
TR|A0A4Q1EPL1|A0A4Q1EPL1_9FLAO -REVSQKYIDSNL---PAEAVTEALLSFIHMTNNNRIGIVNAEESYLLFLVRQTINLIKNY 285
TR|A0A0J7IFG7|A0A0J7IFG7_9FLAO -NKVYENYARSQL---SKNQINEALLSFIHMNNNRLGIINAEESYLLFLIMQTIPLIKNY 285
TR|A0A135WIL0|A0A135WIL0_9FLAO -RNVFASYSQSQL---SSEAIDEALLSFIHMANNRLGIVNAEESYILFLIMCILPLIKKL 284
                                 ..              .     . *::*:  **    . :..  :        : :.
```

Group 41 Alignments

```
NISB                           KIVAN--LGGNLFFLRYTDP-KPHIRLRIKCSDLFLAYGSILEILKR---SRKNRIMSTF 815
MIBB                           RAGIEH-GADRWFFIRYSDTAGQHLRVRFRGEREKLWAGLLPEIGARLVEWQRQGLLAGH 902
TR|A0A2J6NK87|A0A2J6NK87_9STAP DFIDEN-SIQNWHFINYRDP-SEHIRIRILPANEKI-KSKIIYFIKK---LYGEHFIYKY 776
TR|A0A430AMV9|A0A430AMV9_9ENTE KITENM-NINKWFYINYADD-FPHIRFRFVSKEEKH-LNEFVKFLNN---LLQKKYIYNY 494
TR|A0A0R1H5A2|A0A0R1H5A2_LACAM EVLKRAGIQKNCFFIQYNDDGKPSLRLRIWQADERI-LVKILKILNY---LLNIKLINDF 717
TR|A0A809K9G9|A0A809K9G9_LACAI EVLKRAGIRKNYFFIQYNDDEKPSLRLRVWQADEKI-LVSILKILNY---LLHIKLINDF 750
                                   .     . .::.* *     :*.*.           :  :            :  .

NISB                           DISIYDQEVERYGGFDTLELSEAIFCADSKIIPNLLTLIKDTNNDWKVDDVSILV-NYLY 874
MIBB                           ELGQYDPEYERYGGDALAEFTETAFQHDSAAAISLLRLTRRAGFRYTLDEVTAISAA-AL 961
TR|A0A2J6NK87|A0A2J6NK87_9STAP QFEAFNPEINRYGGIENYNNILDYFSISSELAIQRICSND-----TSLEAMTLEI-YYIL 830
TR|A0A430AMV9|A0A430AMV9_9ENTE KLQPFFPEISRYNLFNRGPIVYSIFTYDSQLSSKIFIEEN-----TSNVSDHILL-LYSL 548
TR|A0A0R1H5A2|A0A0R1H5A2_LACAM KLVNFRPEYSRYGGETLFNDALKAFESDSKLALKTINMQE-----EKIKLLTILSAAYTL 772
TR|A0A809K9G9|A0A809K9G9_LACAI KLVNFRAEYSRYGGESLFNDVLKVFECDSNLALKTINMSK-----EKIKLLAILSAVYTF 805
                               .:  :  * .**.           *  .*    .  :          .

NISB                           IIHVHNNRLIGIERDKEKLIYYTLQRLFVSEEYMK----------- 993
MIBB                           LLHMTCNRLIGGSAERERSVLGLARGAVQDN-------LNRRRHLA 1115
TR|A0A2J6NK87|A0A2J6NK87_9STAP IIHMHINRVMGINRNEENKIYCLLANFILSKKYF------------ 932
TR|A0A430AMV9|A0A430AMV9_9ENTE LAHMHMNRASGIDRNFEEKVYSLLYKFVNSKKYIGGFNE------- 655
TR|A0A0R1H5A2|A0A0R1H5A2_LACAM LLHMHFNRFLKPTKKEEYQLNLNILEYEKVKSDRKKYDQNRTRN-- 893
TR|A0A809K9G9|A0A809K9G9_LACAI LLHMHFNRFLKPTQSEERQLNLDMLEYEKVKSDRKKYGQNRTRD-- 926
                               : *:  **      . *  :          :
```

Figure S1. Multiple sequence alignment for representative members of the groups of GLs shown in the SSN in Figure 7 of the main text. NisB and MibB are used as reference sequence and color coding is used to denote key features. Yellow = conserved Tyr in *anti*-GLs or conserved Lys in *syn*-GLs. Pink = conserved Arg residues that bind γ-carboxylate. Blue = conserved Glu found across class I GLs. Green = conserved Arg in *anti*-GLs that acidifies the α-proton of glutamylated Ser/Thr residues for deprotonation or conserved Leu or Ala in class I *syn*-GLs. Red = conserved His for *anti*-GLs.

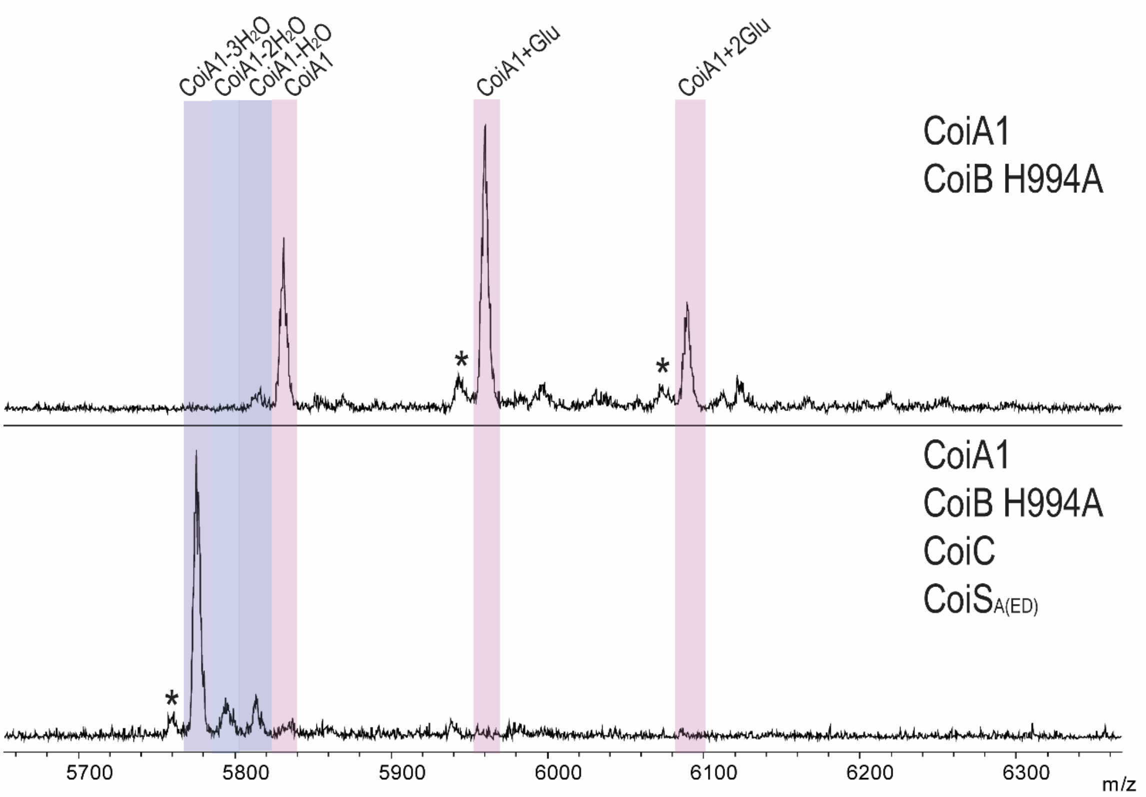

Figure S2. MALDI-TOF MS analysis of co-expression products with CoiB-H994A mutant in the presence and absence of CoiC and $CoiS_{A(ED)}$. Asterisks indicate deamination products that are common during MALDI-TOF MS for peptides of this size. They are not observed in the ESI-MS spectra after GluC-digest.

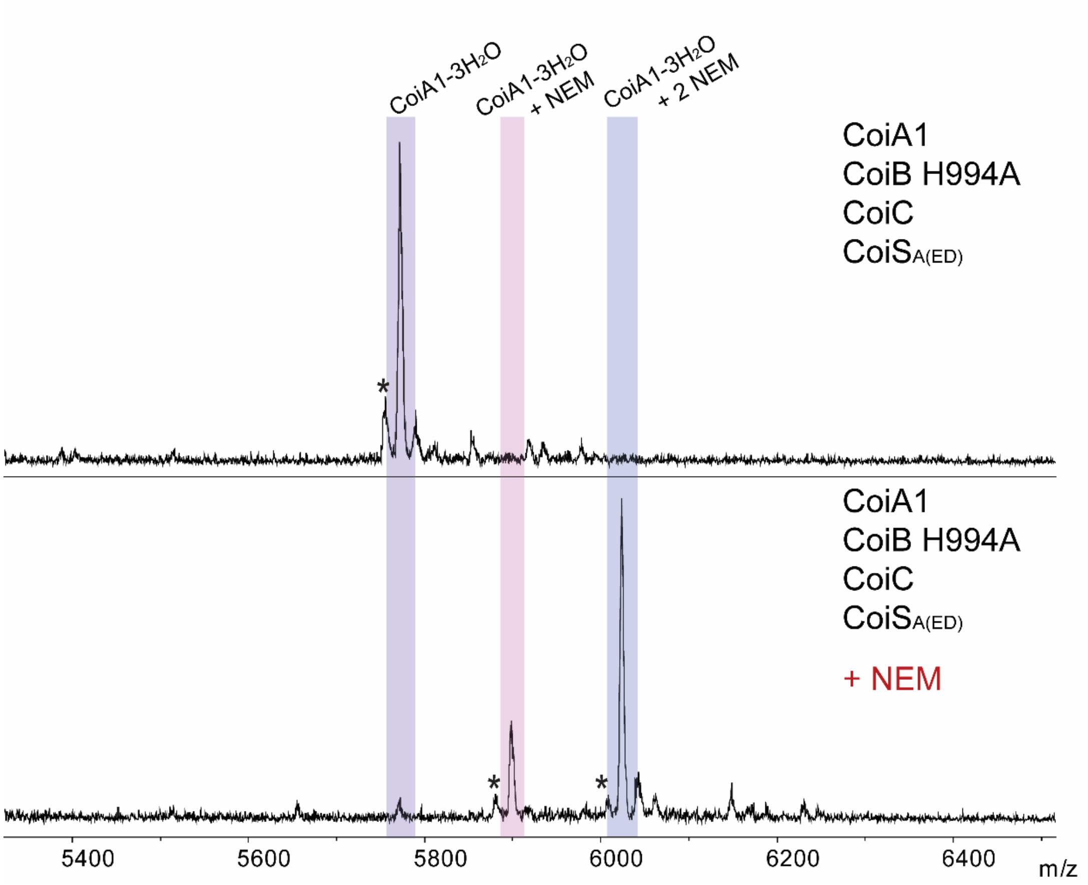

Figure S3. NEM alkylation assay for CoiA1 co-expressed with CoiB-H994A, CoiC, and $CoiS_{A(ED)}$. Asterisks indicate deamination products that are commonly formed during MALDI-TOF MS for peptides of this size.

MALDI-TOF MS, hrMS, and MS/MS characterization of mCoiA1 mutants

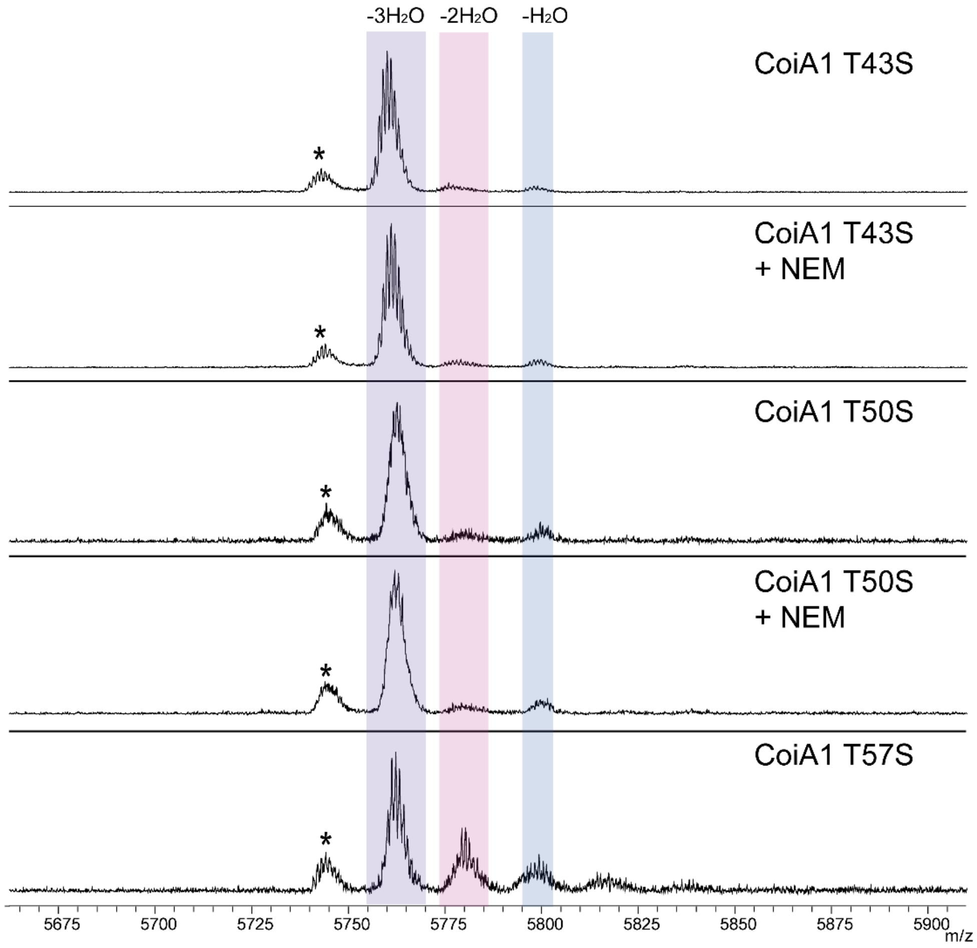

Figure S4. MALDI-TOF MS analysis of CoiA1 mutants co-expressed with CoiB, $CoiS_{A(ED)}$, and CoiC. NEM alkylation assay was performed for mCoiA1-T43S and mCoiA1-T50S. No NEM adducts were observed for these two peptides suggesting the peptides are fully cyclized. Asterisks indicate deamination products that are commonly formed during MALDI-TOF MS for peptides of this size.

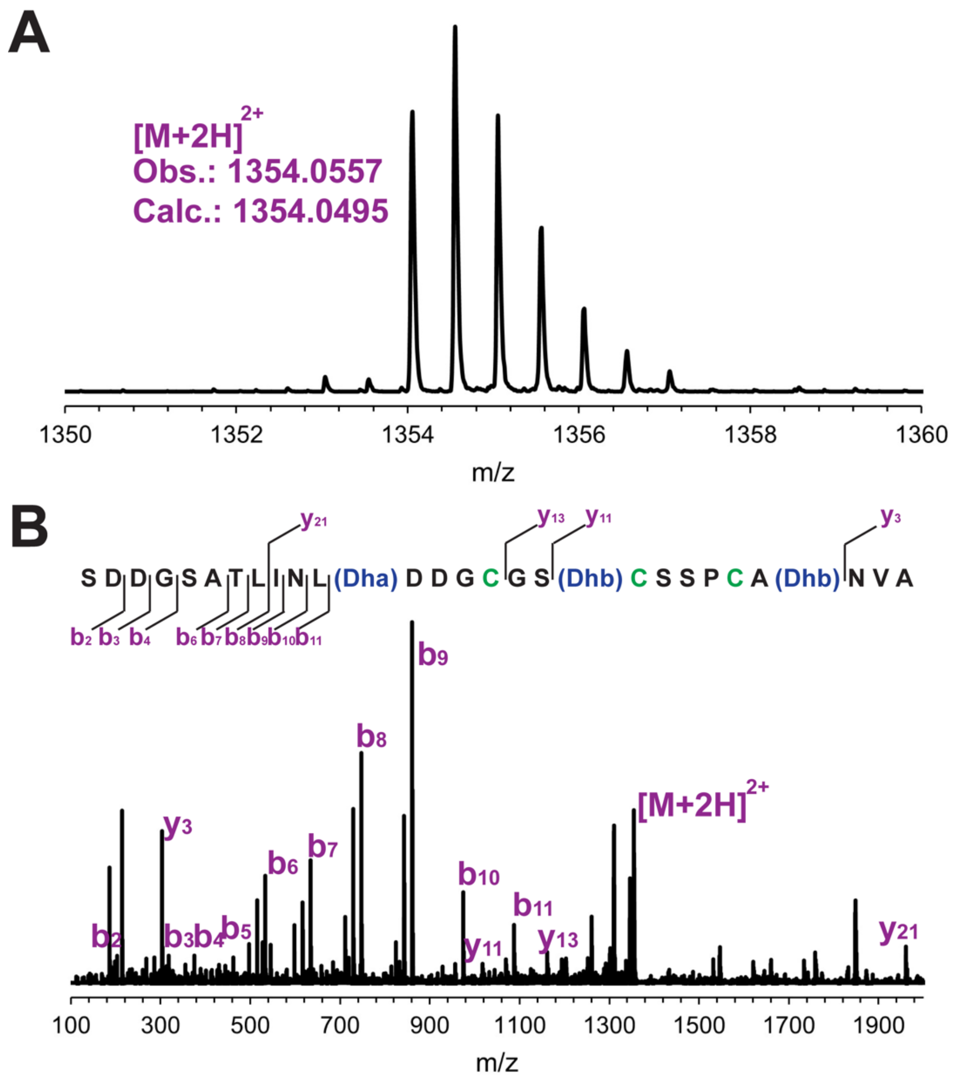

Figure S5. LC-MS analysis of the GluC digestion product of mCoiA1-T43S. A) High-resolution MS analysis. B) Tandem MS analysis. Fragmentation results are consistent with an N-terminal Lan ring and two C-terminal overlapping MeLan rings.

Table S4. Observed and calculated m/z ratios for GluC digestion of mCoiA1 T43S (for annotated ions in **Figure S5**).

Ion	Observed m/z	Calculated m/z	Error (ppm)
b_2	203.0653	203.0662	4.432
y_3	303.1666	303.1663	0.990
b_3	318.0932	318.0932	0.000
b_4	375.1164	375.1147	4.532
b_5	462.1487	462.1467	4.328
b_6	533.1857	533.1838	3.563
b_7	634.2314	634.2315	0.158
b_8	747.3176	747.3155	2.810
b_9	860.4016	860.3996	2.325
b_{10}	974.4433	974.4425	0.821
y_{11}	1017.4126	1017.4128	0.197
b_{11}	1087.5303	1087.5266	3.402
y_{13}	1161.4684	1161.4663	1.808
$[M+2H]^{2+}$	1354.0520	1354.0495	1.846
y_{21}	1960.7864	1960.7834	1.530

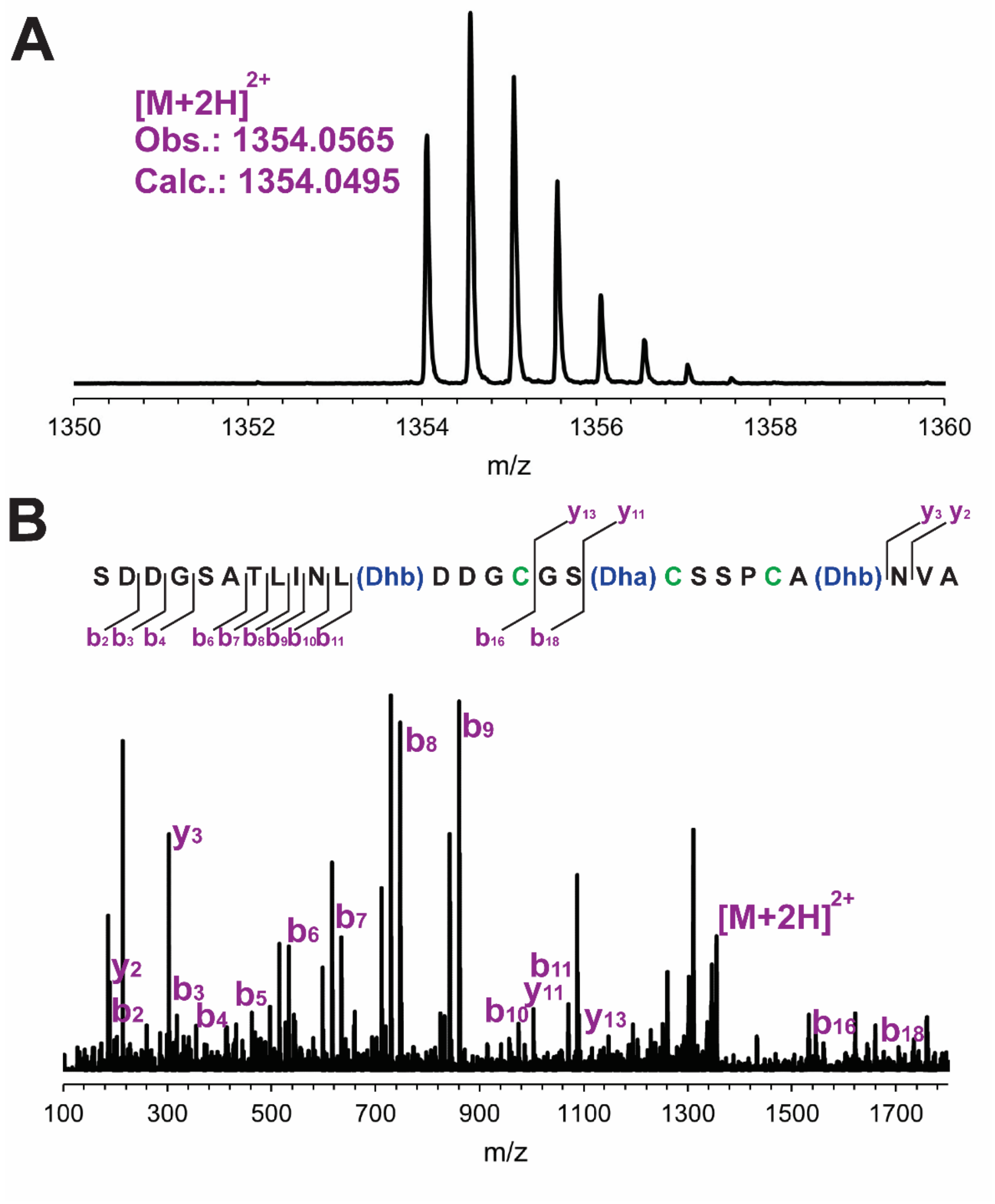

Figure S6. LC-MS analysis of the GluC digestion product of mCoiA1 T50S. A) High-resolution MS analysis. B) Tandem MS analysis. Fragmentation results are consistent with an N-terminal MeLan ring and C-terminal overlapping Lan and MeLan rings.

Table S5. Observed and calculated m/z ratios for GluC digestion of mCoiA1-T50S (for annotated ions in **Figure S6**).

Ion	Observed m/z	Calculated m/z	Error (ppm)
y_2	189.1238	189.1234	2.115
b_2	203.0657	203.0662	2.462
y_3	303.1668	303.1663	1.649
b_3	318.0936	318.0932	1.257
b_4	375.1152	375.1147	1.333
b_5	462.1472	462.1467	1.082
b_6	533.1857	533.1838	3.563
b_7	634.2339	634.2315	3.784
b_8	747.3166	747.3155	1.472
b_9	860.4012	860.3996	1.860
b_{10}	974.4442	974.4425	1.745
y_{11}	1003.3995	1003.3972	2.292
b_{11}	1087.5267	1087.5266	0.092
y13	1147.4559	1147.4507	4.532
$[M+2H]^{2+}$	1354.0505	1354.0495	0.739
b_{16}	1560.6510	1560.6482	1.794
b_{18}	1704.6955	1704.7017	3.637

GC-MS Analysis

Peptides were derivatized for GC-MS experiments according to a previously described procedure using 1 mg of peptide.[1-3] DL- and LL-(Me)Lan standards were prepared as described previously.[1, 4-5]

LC-MS Analysis

Peptides were derivatized with Marfey's reagent according to a procedure described previously using 500 µg of peptide.[1] L-*allo*-D/L- and D-*allo*-D/L-MeLan chemical standards were prepared as previously reported.[1] Reductive desulfurization of mCoiA1 WT was carried out using a procedure previously reported.[1] The protocol for derivatization of L- and D-Ala to generate L- and D-FDAA can be found in the same report.

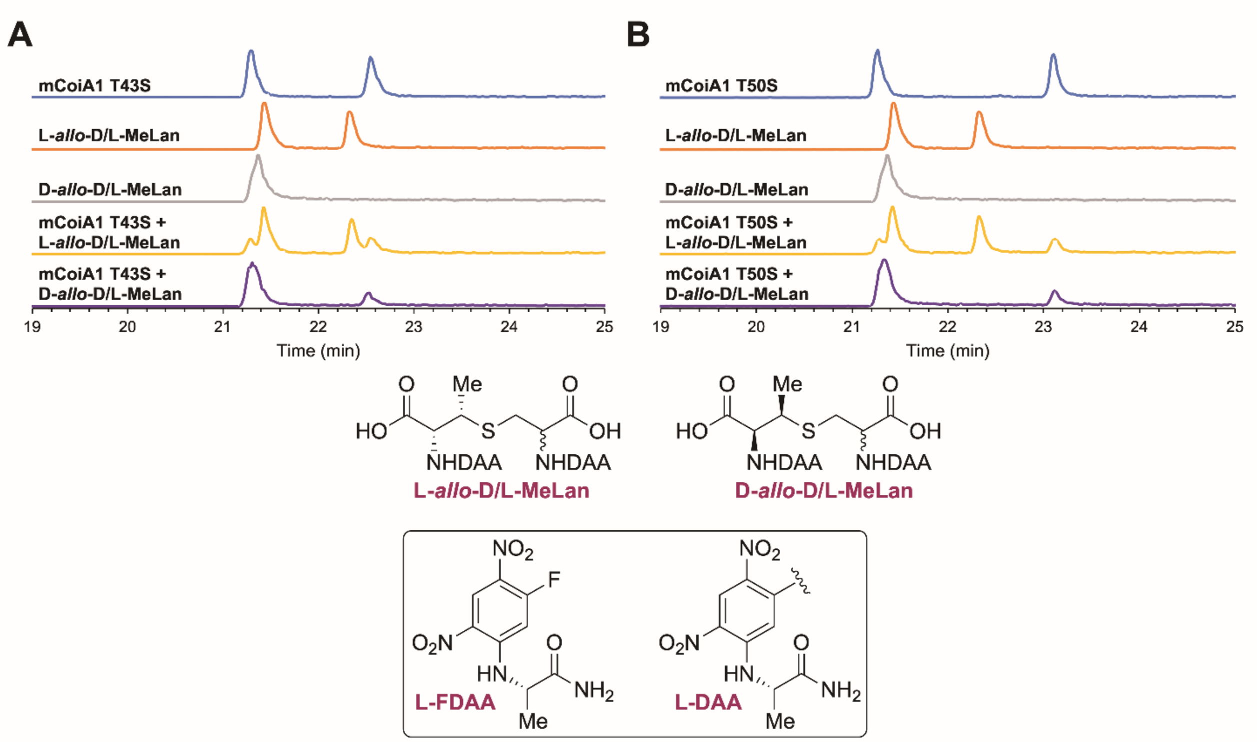

Figure S7. LC-MS analysis of mCoiA1-T43S and mCoiA1-T50S after derivatization of the hydrolysates with L-FDAA. Structures of derivatized standards are shown at the bottom of the figure. Extracted ion chromatogram (EIC) monitored for bisderivatized MeLan (m/z = 727.1742). A) Analysis of mCoiA1 T43S with co-injections confirming the presence of D-*allo*-D/L-MeLan. B) Analysis of mCoiA1 T50S with co-injections confirming the presence of D-*allo*-D/L-MeLan.

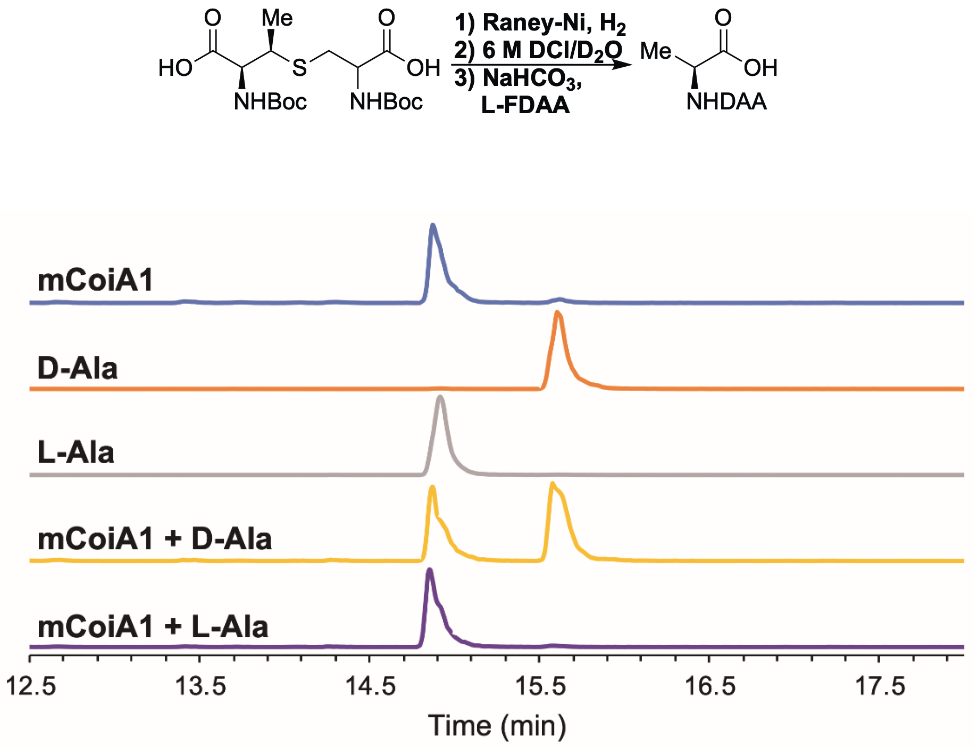

Figure S8. Analysis of Ala residues after treating mCoiA1 with 6 M DCl/D_2O, followed by Boc-protection to yield $(Boc)_2$-MeLan. $(Boc)_2$-MeLan was isolated by LC and desulfurized using Raney-Ni and H_2, followed by Boc-deprotection, and derivatization with L-FDAA. EIC monitored for Ala (m/z = 342.1044). Co-injections confirm the presence of L-Ala.

Genome Neighborhood Analysis of Glutamyl Lyase SSN

Table S6. Genome neighborhood analysis of each group from GL SSN from **Figure 7**. BGC components that are annotated that are likely found as class defining enzymes are listed for each group. Annotated nitroreductases are likely azoline oxidoreductases that convert azolines to azoles. For a discussion of the function of YcaO proteins, see reference [6]

Group	Split or Full length LanB	BGC components
1	Full length	Class I lanthipeptide BGC containing full length LanB and LanC
2	1 GL stand alone (or fused to methyltransferase) & 1 full length	Class I lanthipeptide BGCs containing two GL domains (full length LanB & extra GL domain either stand alone or fused to methyltransferase). LanC cyclase also present. Coi-like BGCs
3	Split	Split LanB, pyridine synthase, YcaO, nitroreductase
4	Split	Multiple YcaO, nitroreductase, split LanB
5	Split	Split LanB, pyridine synthase, YcaO, nitroreductase
6	Split	Split LanB, pyridine synthase, YcaO, nitroreductase
7	Split	Split LanB, nitroreductase containing fused GL domain, pyridine synthase, and YcaO. Similar in architecture to lactazole BGC

**8**	Split	Split LanB, nitroreductase containing fused GL domain, pyridine synthase, and YcaO. Similar in architecture to lactazole BGC
**9**	Split	Split LanB, dehydrogenase containing fused GL domain, pyridine synthase, and YcaO. Similar in architecture to lactazole BGC
**10**	Split (sometimes have additional stand-alone GL domain or fused to methyltransferase)	Class I lanthipeptide BGCs containing split LanB and LanC. In some cases, Coi-like BGCs are observed (extra stand-alone GL domain or fused to methyltransferase)
**11**	Split	YcaO, nitroreductase, split LanB
**12**	Split	YcaO, nitroreductase, split LanB
**13**	Split	YcaO, nitroreductase, split LanB
**14**	Split	Class I lanthipeptide BGC containing split LanB and LanC
**15**	Split	YcaO, split LanB
**16**	Split	YcaO, nitroreductase, split LanB
**17**	Split or full length	Split (or full length) LanB, nitroreductase, pyridine synthase, and YcaO
**18**	Split	YcaO (one or two), nitroreductase, split LanB
**19**	Split	Class I lanthipeptide BGC containing split LanB and LanC
**20**	Split	YcaO, split LanB
**21**	Split	Split LanB and nitroreductase
**22**	Split	YcaO, nitroreductase, split LanB
**23**	Split	Class I lanthipeptide BGC containing split LanB and LanC
**24**	Split	Split LanB, nitroreductase, pyridine synthase, and YcaO. Likely making thiopeptides
**25**	Split	Split LanB, LanC (sometimes), YcaO, nitroreductase
**26**	Split	YcaO, nitroreductase, split LanB
**27**	Split	YcaO, nitroreductase, split LanB
**28**	Split	1 YcaO, 1 nitroreductase, 1 split LanB, pyridine synthase
**29**	Split	Split LanB, nitroreductase, pyridine synthase, and YcaO
**30**	Split	YcaO, nitroreductase, split LanB
**31**	Stand alone elimination domain	Stand alone elimination domain
**32**	Split	Split LanB, nitroreductase, pyridine synthase, and YcaO
**33**	Split	Split LanB (sometimes have two GL domains)
**34**	Split	Split LanB, nitroreductase, pyridine synthase, and multiple YcaO
**35**	Split	Split LanB, nitroreductase, pyridine synthase, and YcaO
**36**	Split	Split LanB, nitroreductase, pyridine synthase, and YcaO
**37**	Split	Split LanB, nitroreductase, pyridine synthase, and YcaO
**38**	Split	Split LanB, nitroreductase, pyridine synthase, and YcaO
**39**	Split	Split LanB, nitroreductase, pyridine synthase, and YcaO
**40**	Full length	Full length LanB
**41**	Full length	Class I lanthipeptide BGC containing full length LanB and LanC
**42**	Split	Split LanB, nitroreductase containing fused GL domain, pyridine synthase, and YcaO. Similar in architecture to lactazole BGC

**43**	Split	Split LanB, nitroreductase containing fused GL domain, pyridine synthase, and YcaO. Similar in architecture to lactazole BGC
**44**	Split	Two YcaO, nitroreductase, split LanB. Likely making LAPs
**45**	Split	Split LanB
**46**	Split	Split LanB and YcaO
**47**	Split	YcaO, nitroreductase, split LanB. Likely making LAPs
**48**	Split	YcaO, nitroreductase, split LanB. Likely making LAPs
**49**	Full length	1 full length LanB only
**50**	Stand alone elimination domain	Stand alone elimination domain
**51**	Stand alone elimination domain	Stand alone elimination domain
**52**	Stand alone elimination domain	Stand alone elimination domain
**53**	Stand alone elimination domain	Stand alone elimination domain with LanC. No glutamylation enzyme
**54**	Split	Split LanB and radical SAM synthase
**55**	Stand alone elimination domain	Stand alone elimination domain
**56**	Split	Split LanB, nitroreductase, pyridine synthase, and YcaO

Bioinformatic Analysis of OlvA and CoiA1 precursor peptides

OlvA and CoiA1 precursor peptides were used as query for BLAST (Basic Local Alignment Search Tool) using PSI-BLAST (Position-Specific Iterated BLAST) for the program algorithm. PSI-BLAST threshold used was 0.005. Only peptide sequences above threshold are used for analysis. This results in identification of 43 hits for OlvA and 301 hits for CoiA1. A sequence LOGO was generated for each data set to identify conserved sequences (**Figure 8B**). A description table is provided below for each data set (**Tables S7 and S8**).

Table S7. Description table of orthologs obtained from PSI-BLAST of OlvA

Scientific Name	Total Score	Query Cover	E value	Per. ident	Acc. Len	Accession
Streptomyces olivaceus	110	100%	4.00E-30	98.21	58	WP_195888158.1
Streptomyces olivaceus	101	100%	1.00E-26	91.07	58	WP_224289644.1
Streptomyces coelicoflavus	99.8	100%	6.00E-26	87.5	58	WP_108987711.1
Streptomyces violaceoruber	92.4	100%	4.00E-23	81.67	62	WP_030869849.1
Streptomyces anthocyanicus	92.4	100%	5.00E-23	81.67	60	WP_189285448.1
Streptomyces toyocaensis	92	96%	6.00E-23	81.48	58	WP_107061326.1
Streptomyces anthocyanicus	90.9	100%	1.00E-21	80	139	GGL83324.1
Streptomyces	88.6	100%	2.00E-21	76.67	62	WP_197924123.1
Streptomyces olivaceus	81.6	73%	5.00E-19	97.56	44	WP_194275403.1
Streptomyces sp. SID8367	80.5	92%	3.00E-18	73.08	58	WP_202455230.1
Streptomyces sp. PsTaAH-137	80.5	92%	3.00E-18	73.08	59	WP_220093178.1
Streptomyces sp. SID8367	80.1	92%	3.00E-18	73.08	57	MYT71546.1

Streptomyces sp. NA03103	75.5	71%	1.00E-16	90	40	WP_176156416.1
Streptomyces sp. RM72	75.5	73%	2.00E-16	87.8	42	WP_210976197.1
unclassified Streptomyces	73.2	71%	1.00E-15	85	40	WP_210917375.1
Streptomyces sp. TSRI0107	68.2	100%	2.00E-13	58.93	56	WP_107468502.1
Streptomyces sp. SLBN-134	62.4	75%	3.00E-11	71.43	58	WP_222126044.1
Streptomyces nodosus	59.3	78%	6.00E-10	61.36	56	WP_107070452.1
Streptomyces sp. BJ20	58.9	100%	8.00E-10	62.5	55	WP_244792597.1
unclassified Streptomyces	58.9	98%	8.00E-10	52.73	55	WP_107646905.1
Actinospica acidiphila	57	100%	4.00E-09	60.71	55	NEA81815.1
unclassified Streptomyces	55.5	87%	2.00E-08	67.35	57	WP_107052129.1
Streptomyces sp. T12	53.9	87%	8.00E-08	65.31	57	WP_145830614.1
Streptomyces	50.4	71%	2.00E-06	55	49	WP_103503400.1
Streptomyces carminius	49.7	71%	3.00E-06	52.5	49	WP_100202448.1
Streptomyces phaeolivaceus	49.3	71%	4.00E-06	52.5	49	WP_152170413.1
Streptomyces griseostramineus	48.9	78%	6.00E-06	59.09	55	WP_184829510.1
Actinobacteria bacterium OK074	48.9	69%	9.00E-06	51.28	63	KPI02188.1
unclassified Streptomyces	48.1	64%	1.00E-05	52.78	41	WP_078968209.1
Streptomyces sp. WAC 06738	48.1	64%	1.00E-05	55.56	58	AZM47828.1
Streptomyces sp. GMR22	48.1	94%	2.00E-05	44.44	62	MBA6440663.1
Streptomyces sp. DASNCL29	47.4	85%	4.00E-05	51.02	62	WP_138915795.1
Streptomyces rhizosphaericus	47	85%	4.00E-05	51.02	62	WP_164429130.1
Streptomyces sp. CBMA29	46.6	64%	5.00E-05	55.56	57	WP_188281634.1
Streptomyces sp. MNP-20	45.4	85%	1.00E-04	51.02	50	WP_172387339.1
Streptomyces sp. WAC05858	45.8	89%	2.00E-04	47.06	76	WP_125755668.1
Streptomyces endocoffeicus	44.3	89%	5.00E-04	45.1	64	WP_201858267.1
Streptomyces sp. 11-1-2	44.3	89%	8.00E-04	45.1	76	WP_119994418.1
Streptomyces sp. 11-1-2	44.7	89%	9.00E-04	45.1	107	ASQ94458.1
Streptomyces aurantiogriseus	43.9	100%	0.001	43.1	67	WP_189934944.1
Streptomyces sp. AC602_WCS936	42.7	67%	0.002	73.68	61	WP_217247104.1
Thermocatellispora tengchongensis	42.4	83%	0.003	50	59	WP_185056813.1
Planomonospora sp. ID82291	42	67%	0.004	51.28	62	WP_230885174.1

Table S8. Description table of orthologs obtained from PSI-BLAST of CoiA1

Scientific Name	Total Score	Query Cover	E value	Per. ident	Acc. Len	Accession
Streptomyces	117	100%	7.00E-33	100	59	WP_011031313.1
Streptomyces coelicolor	112	96%	8.00E-31	98.25	57	WP_154871743.1
Streptomyces spongiicola	101	100%	1.00E-26	86.44	59	WP_109296521.1
Streptomyces	99	100%	1.00E-25	84.75	59	WP_054100455.1
Streptomyces	97.4	100%	5.00E-25	83.05	59	WP_016325332.1
Streptomyces sp. ADI98-12	97.1	100%	6.00E-25	83.05	59	WP_124288031.1
Streptomyces sp. SS06011	97.1	100%	7.00E-25	83.05	59	WP_186778934.1
Streptomyces diastaticus	96.3	100%	2.00E-24	81.36	59	WP_102927693.1
Streptomyces	95.9	100%	2.00E-24	83.05	59	WP_007491157.1
Streptomyces alfalfae	95.5	98%	3.00E-24	82.76	60	WP_198504430.1
Streptomyces sp. NBRC 110030	95.1	100%	4.00E-24	81.36	59	WP_055468657.1
Streptomyces	94.4	100%	1.00E-23	81.36	59	WP_108990580.1
Streptomyces anthocyanicus	94	100%	1.00E-23	81.36	59	WP_189283454.1
Streptomyces parvus	92.8	100%	3.00E-23	77.97	59	WP_164206150.1
unclassified Streptomyces	92.8	100%	3.00E-23	77.97	59	WP_093542814.1
Streptomyces sp. SID6013	88.6	94%	2.00E-21	80.36	57	MYS54847.1
Streptomyces olivaceus	86.7	100%	1.00E-20	74.58	59	WP_194274770.1
Streptomyces venezuelae	86.3	100%	1.00E-20	71.19	59	WP_150220421.1
Streptomyces aureocirculatus	84	100%	1.00E-19	71.19	59	WP_030570919.1
Streptomyces flavofungini	84	100%	1.00E-19	74.58	59	WP_190118691.1
Streptomyces chartreusis	82	98%	6.00E-19	72.41	58	WP_176576428.1
Streptomyces albidoflavus	78.6	100%	1.00E-17	69.49	58	WP_085478286.1
Streptomyces sp. AK010	78.6	100%	1.00E-17	71.19	58	WP_185006854.1
Streptomyces	78.2	67%	2.00E-17	97.5	43	WP_007491158.1
Streptomyces	77.8	67%	2.00E-17	97.5	43	WP_011031314.1
Streptomyces sp. WAC02707	77	67%	4.00E-17	97.5	43	WP_125768841.1
Streptomyces sp. NBRC 110611	76.3	67%	1.00E-16	92.5	43	WP_079124468.1
Streptomyces sp. TSRI0384-2	75.9	67%	1.00E-16	95	43	WP_100458265.1
Streptomyces	75.9	67%	1.00E-16	95	43	WP_076683627.1
Streptomyces	73.9	67%	7.00E-16	92.5	43	WP_079060774.1
Streptomyces sp. Tu 3180	73.9	67%	8.00E-16	92.5	43	WP_159528270.1
Streptomyces venezuelae	73.9	67%	9.00E-16	92.5	43	WP_150220422.1
Streptomyces	73.6	67%	9.00E-16	90	43	WP_093542812.1
Streptomyces flavofungini	73.6	67%	1.00E-15	92.5	43	WP_190118690.1
Streptomyces anthocyanicus	73.2	67%	2.00E-15	92.5	43	WP_189283455.1
Streptomyces paludis	72.8	67%	2.00E-15	92.5	43	WP_114658459.1

Streptomyces sp. B15	72.4	67%	3.00E-15	92.5	43	WP_210966112.1
Streptomyces sp. NBRC 110030	71.6	67%	7.00E-15	87.5	43	WP_063788268.1
Streptomyces olivaceus	70.9	67%	1.00E-14	90	43	WP_194274769.1
Streptomyces sp. AK010	70.1	67%	3.00E-14	87.5	43	WP_185006852.1
Streptomyces sp. NBRC 110611	67.4	100%	4.00E-13	59.32	59	WP_066938278.1
Streptomyces malaysiensis	67	76%	7.00E-13	72.92	65	WP_079257514.1
Streptomyces albidoflavus	66.2	67%	7.00E-13	85	43	WP_085478287.1
Actinomadura lepetitiana	66.2	67%	1.00E-12	85	43	NDU75967.1
Streptomyces	65.9	67%	1.00E-12	80	42	WP_232564462.1
Actinomadura lepetitiana	65.9	67%	1.00E-12	82.5	49	NDU73750.1
Streptomyces sp. GbtcB7	65.1	67%	2.00E-12	82.5	43	WP_217572510.1
Actinomadura viridis	65.1	67%	3.00E-12	82.5	43	WP_231403590.1
Streptomyces daliensis	64.3	67%	6.00E-12	80	46	MBR7676590.1
Pseudonocardia kunmingensis	64.3	67%	6.00E-12	75	56	WP_170231817.1
Streptomyces sp. MnatMP-M27	63.5	67%	8.00E-12	77.5	42	SCF82208.1
Streptomyces sp. MnatMP-M27	64.3	76%	8.00E-12	70.83	65	WP_093701263.1
Streptomyces sp. B15	62.4	81%	3.00E-11	66.67	52	WP_210966111.1
Streptomyces sp. JJ66	62	67%	4.00E-11	75	43	WP_219049695.1
Actinomadura bangladeshensis	62.4	72%	4.00E-11	69.77	58	WP_163062238.1
Streptomyces aureocirculatus	61.6	57%	5.00E-11	88.24	34	WP_078965644.1
Actinomadura sp. CNU-125	62	98%	5.00E-11	51.72	59	OLT34221.1
Actinomadura sp. 7K534	61.6	72%	8.00E-11	69.77	58	WP_132045422.1
Actinomadura algeriensis	61.2	71%	1.00E-10	69.05	58	WP_225961189.1
Actinomadura bangladeshensis	60.8	72%	2.00E-10	67.44	58	WP_131943984.1
Sphaerisporangium album	60.5	59%	2.00E-10	80	48	WP_114033294.1
Actinomadura montaniterrae	59.7	81%	5.00E-10	61.54	58	WP_151538273.1
Yinghuangia sp. ASG 101	59.3	64%	5.00E-10	73.68	54	WP_231777716.1
Actinomadura sp. WAC 06369	59.3	71%	8.00E-10	66.67	58	WP_125618143.1
Yinghuangia sp. KLBMP8922	58.9	67%	8.00E-10	72.5	50	WP_235055820.1
Candidatus Frankia californiensis	58.5	98%	1.00E-09	53.45	59	SBW22517.1
Streptomyces sp. NRRL B-24572	57.8	67%	2.00E-09	70	44	WP_086824825.1
Frankia canadensis	55.5	61%	2.00E-08	72.22	57	WP_243407251.1
Streptomyces sp. RTd22	54.7	100%	5.00E-08	46.88	64	WP_063734380.1
Streptomyces lydicamycinicus	52.8	67%	2.00E-07	67.5	48	GAO08700.1
Streptomyces	52.4	67%	3.00E-07	67.5	46	WP_078885829.1
Streptomyces albulus	52.4	86%	4.00E-07	56.86	59	WP_189867673.1
Micromonospora	51.6	84%	6.00E-07	64	52	WP_187692502.1
Streptomyces sp. RFCAC02	51.6	86%	9.00E-07	49.09	61	WP_240796446.1
Actinomycetia bacterium	51.2	100%	1.00E-06	45.59	69	MCA1672664.1

Micromonospora coxensis	50.8	76%	1.00E-06	67.39	52	WP_088974495.1
Actinoalloteichus spitiensis	51.2	91%	2.00E-06	46.43	83	WP_016698392.1
Streptomyces	50.4	79%	2.00E-06	59.57	59	WP_006383508.1
Actinomadura chibensis	50.1	79%	2.00E-06	53.19	53	WP_083981449.1
Streptomyces	50.4	86%	2.00E-06	54.9	59	WP_014143559.1
Streptosporangium amethystogenes	50.1	96%	3.00E-06	52.63	54	WP_084195647.1
Micromonospora globbae	50.1	77%	3.00E-06	63.04	52	WP_120331046.1
Streptomyces aidingensis	50.1	96%	3.00E-06	42.62	63	SFD65031.1
Micromonospora gifhornensis	49.7	76%	4.00E-06	64.44	52	WP_204292814.1
Micromonospora sp. C32	49.7	84%	4.00E-06	62	52	WP_210942429.1
Micromonospora purpureochromogenes	49.7	77%	4.00E-06	63.04	52	WP_088962583.1
Streptomyces radicis	49.7	81%	4.00E-06	60.42	59	WP_120700216.1
Micromonospora sp. D93	49.7	77%	4.00E-06	63.04	52	WP_210819252.1
Micromonospora	49.7	77%	4.00E-06	63.04	52	WP_018829768.1
Micromonospora sp. NBRC 110037	49.3	84%	6.00E-06	62	52	WP_082930958.1
Streptomyces coelicoflavus	49.3	100%	7.00E-06	51.61	60	WP_108989980.1
Micromonospora purpureochromogenes	48.9	83%	8.00E-06	63.27	52	WP_084213113.1
Micromonospora	48.9	79%	8.00E-06	61.7	52	WP_104113317.1
Nonomuraea sp. TT08I-71	48.5	79%	1.00E-05	57.45	52	GHJ52883.1
Micromonospora narathiwatensis	48.5	74%	1.00E-05	63.64	52	WP_091191932.1
Micromonospora chaiyaphumensis	48.1	81%	1.00E-05	56.25	52	WP_091270023.1
Nocardiopsis sp. CNT312	48.5	86%	1.00E-05	49.02	57	WP_081748662.1
Streptomyces qinzhouensis	48.5	94%	1.00E-05	40.98	64	WP_146482433.1
Microbispora	48.1	77%	2.00E-05	48.98	58	WP_142567707.1
Nocardiopsis halotolerans	48.1	74%	2.00E-05	54.55	52	WP_017573280.1
Streptomyces malaysiensis	48.1	86%	2.00E-05	54.9	58	WP_167503187.1
Catenulispora sp.	48.1	79%	2.00E-05	53.19	56	NUR25381.1
Streptomyces ipomoeae	48.1	100%	2.00E-05	49.18	59	WP_009337871.1
Nocardiopsis sp. RV163	47.8	64%	2.00E-05	60.53	52	WP_152691982.1
Streptomyces monomycini	47.8	100%	2.00E-05	48.44	59	WP_030023383.1
Streptomyces aidingensis	47.8	81%	3.00E-05	48.08	55	WP_245834535.1
Streptomyces neyagawaensis	47.8	91%	3.00E-05	49.09	59	WP_055539243.1
Streptomyces albidochromogenes	47.8	86%	3.00E-05	54.9	60	WP_138900807.1
Streptomyces antioxidans	47.8	98%	3.00E-05	50	60	WP_046089400.1
Streptomyces sp. 3213.3	47.4	67%	3.00E-05	65	55	WP_093622567.1
Streptomyces	47.4	100%	3.00E-05	52.46	58	WP_189927986.1
Nocardiopsis chromatogenes	47.4	67%	3.00E-05	52.5	50	WP_017623422.1

Streptomyces scabiei	47.4	62%	4.00E-05	62.16	60	WP_037699644.1
Streptomyces monomycini	47.4	76%	4.00E-05	48.98	53	WP_238783698.1
Streptomyces monomycini	47	76%	4.00E-05	48.98	53	WP_208870101.1
Streptomyces sp. CNQ085	47.4	98%	4.00E-05	46.55	59	WP_241714151.1
Streptomyces sp. L2	47.4	81%	4.00E-05	56.25	59	WP_129307262.1
Streptomyces sp. TLI_185	47	86%	4.00E-05	56.86	57	WP_123970184.1
Streptomyces sp. MBT49	47	86%	4.00E-05	56.86	56	WP_200725131.1
Nocardiopsis sp. FR6	47	74%	4.00E-05	52.27	52	WP_159944683.1
Streptomyces sp. LaPpAH-108	47	79%	4.00E-05	57.45	59	WP_018543778.1
Streptomyces purpureus	47	86%	4.00E-05	54.9	59	WP_189203508.1
Streptomyces	47	100%	5.00E-05	48.44	59	WP_030630840.1
unclassified Streptomyces	47	100%	5.00E-05	48.33	58	WP_215158055.1
Streptomyces xylophagus	47	83%	5.00E-05	59.18	59	WP_043680782.1
Streptomyces chrestomyceticus	47	100%	5.00E-05	48.39	59	WP_206507371.1
Streptomyces	47	86%	5.00E-05	54.9	59	WP_030750825.1
Streptomyces sp. W18L9	47	67%	5.00E-05	55	61	WP_181801456.1
Streptomyces sp. PTM05	47	100%	5.00E-05	42.37	57	WP_222982355.1
Streptomyces uncialis	47	81%	6.00E-05	54.17	59	WP_079185188.1
Streptomyces microflavus subgroup	47	79%	6.00E-05	57.45	59	WP_015612045.1
Streptomyces sp. SID6648	46.6	79%	6.00E-05	55.32	59	NED00557.1
Streptomyces sp. WM6386	46.6	81%	7.00E-05	54.17	59	WP_046259642.1
unclassified Streptomyces	46.6	81%	7.00E-05	54.17	59	WP_019982949.1
Streptomyces sp. CBMA156	46.2	89%	8.00E-05	49.06	53	WP_188299519.1
Streptomyces ipomoeae	46.6	77%	8.00E-05	47.17	60	WP_009322874.1
Streptomyces curacoi	46.6	81%	8.00E-05	54.17	59	WP_062145163.1
Streptomyces viridochromogenes	46.2	81%	9.00E-05	54.17	59	WP_004000001.1
Streptomyces sp. MW-W600-10	46.6	81%	9.00E-05	54.17	63	WP_218525985.1
Streptomyces sp. AVP053U2	46.6	64%	9.00E-05	52.63	67	WP_079139587.1
Streptomyces clavuligerus	46.6	96%	9.00E-05	38.71	64	WP_009996537.1
Streptomyces sp. MA3_2.13	46.2	79%	1.00E-04	57.45	59	WP_228081330.1
Frankia sp. AiPa1	46.2	74%	1.00E-04	52.27	50	MCK9883265.1
Streptomyces sp. AVP053U2	46.2	64%	1.00E-04	52.63	63	ODA69368.1
Streptomyces sp. MST-110588	46.2	67%	1.00E-04	62.5	58	WP_242585209.1
Streptomyces sp. SID13726	46.2	81%	1.00E-04	54.17	59	WP_164434404.1
Streptomyces sp. PT12	46.2	64%	1.00E-04	52.63	63	WP_113690260.1
Streptomyces	46.2	81%	1.00E-04	54.17	59	WP_016825826.1
Streptomyces cellulosae	45.8	67%	1.00E-04	65	56	GHE73883.1
Streptomyces	46.2	81%	1.00E-04	54.17	59	WP_030642794.1
Streptomyces sp. ISL-12	46.2	79%	1.00E-04	55.32	59	WP_215170566.1

Streptomyces pactum	45.8	91%	1.00E-04	50	59	WP_055419570.1
Streptomyces jumonjinensis	46.2	94%	1.00E-04	39.34	64	MQT04812.1
Streptomyces	45.8	81%	1.00E-04	54.17	59	WP_018489256.1
Streptomyces sp. RG38	45.8	81%	1.00E-04	54.17	59	WP_210875011.1
Streptomyces tsukubensis	46.2	94%	1.00E-04	39.34	64	WP_006345935.1
Streptomyces inusitatus	45.8	81%	1.00E-04	52.08	59	WP_190122385.1
Streptomyces cyaneus	45.8	81%	1.00E-04	54.17	59	WP_128430927.1
Streptomyces albus	45.8	79%	1.00E-04	55.32	59	WP_030546349.1
unclassified Streptomyces	45.4	81%	1.00E-04	52.08	47	WP_241662484.1
Streptomyces sp. MNU89	45.8	81%	1.00E-04	54.17	59	WP_230241243.1
Streptomyces	45.8	81%	1.00E-04	52.08	59	WP_078902216.1
Microbispora amethystogenes	45.8	74%	1.00E-04	47.83	57	WP_204283909.1
Catellatospora coxensis	45.8	77%	2.00E-04	54.35	54	WP_203694375.1
Streptomyces sp. OfavH-34-F	45.8	100%	2.00E-04	49.18	59	WP_239221654.1
Allocatelliglobosispora scoriae	45.8	86%	2.00E-04	45.1	55	WP_184840288.1
Alloactinosynnema sp. L-07	45.4	74%	2.00E-04	54.55	53	WP_231949584.1
Streptomyces sp. NRRL F-5053	45.4	81%	2.00E-04	46.15	55	WP_234431160.1
Streptomyces sp. YIM 98790	45.1	64%	2.00E-04	52.63	42	WP_207957328.1
Microbispora	45.8	94%	2.00E-04	47.54	63	WP_111699036.1
Streptomyces glomeratus	45.4	100%	2.00E-04	42.37	57	WP_234518395.1
Streptomyces anthocyanicus	47	67%	2.00E-04	62.5	134	GHC27930.1
Streptomyces sp. AC602_WCS936	45.4	100%	2.00E-04	49.18	59	WP_217247095.1
Streptomyces albus	45.4	71%	2.00E-04	50	53	WP_173874729.1
Candidatus Nocardiopsis merdipullorum	45.4	64%	2.00E-04	57.89	55	HIY40925.1
Streptomyces monomycini	45.4	64%	2.00E-04	50	61	WP_208870183.1
Nocardiopsis deserti	45.1	64%	2.00E-04	52.63	52	WP_150253403.1
Streptomyces roseirectus	45.4	66%	2.00E-04	53.85	60	WP_187751885.1
Streptomyces	45.4	93%	2.00E-04	40.68	63	WP_030408389.1
Streptomyces sp. b94	45.4	81%	2.00E-04	56.25	59	WP_210964678.1
Streptomyces sp. CB02414	45.4	100%	2.00E-04	49.18	59	WP_073732281.1
Streptomyces sp. HUCO-GS316	45.4	77%	2.00E-04	47.83	60	WP_202424667.1
Streptomyces griseoaurantiacus	45.4	94%	2.00E-04	43.1	60	WP_191854063.1
Streptomyces smaragdinus	45.1	67%	3.00E-04	62.5	57	WP_153450700.1
Streptomyces actuosus	45.1	67%	3.00E-04	60	58	WP_205382920.1
Streptomyces marianii	45.1	67%	3.00E-04	60	58	TLQ46066.1
Microbispora	45.1	74%	3.00E-04	46.81	58	WP_150935661.1
Streptomyces marianii	45.1	67%	3.00E-04	60	54	WP_234042566.1
Nocardiopsis sp. CNS-639	45.1	64%	3.00E-04	52.63	52	WP_019610919.1
Streptomyces sp. HUCO-GS316	45.1	77%	3.00E-04	47.83	56	MXM62147.1

Streptomyces anthocyanicus group	45.1	67%	3.00E-04	62.5	60	WP_164272930.1
Streptomyces sp. AC495_CC817	45.1	83%	3.00E-04	52	59	WP_217181757.1
Streptomyces acidiscabies	45.1	77%	3.00E-04	45.65	60	WP_075662580.1
Streptomyces sp. NA02950	45.1	64%	3.00E-04	50	63	WP_176187576.1
Streptomyces arenae	44.7	67%	4.00E-04	62.5	59	MCG7202628.1
Streptomyces fulvoviolaceus	44.7	67%	4.00E-04	62.5	59	WP_030609485.1
Streptomyces ipomoeae	44.7	81%	4.00E-04	54.17	59	WP_141573377.1
unclassified Frankia	45.1	61%	4.00E-04	55.56	69	WP_239310792.1
unclassified Frankia	44.7	61%	4.00E-04	52.78	64	WP_054571360.1
Streptomyces jumonjinensis	45.4	59%	4.00E-04	54.29	84	WP_153526123.1
Actinomadura rudentiformis	44.7	62%	4.00E-04	51.35	61	WP_151565208.1
Streptomyces zagrosensis	44.7	81%	4.00E-04	52.08	57	WP_184568501.1
Frankia sp. Allo2	44.7	93%	5.00E-04	40	61	WP_051875411.1
Streptomyces jumonjinensis	44.7	81%	5.00E-04	54.17	59	WP_153525006.1
Nocardiopsis kunsanensis	44.3	64%	5.00E-04	57.89	55	WP_204366767.1
Nocardiopsis sp. MG754419	44.3	86%	5.00E-04	47.06	53	WP_211715620.1
Streptomyces sp. 2112.2	44.3	100%	5.00E-04	44.07	57	WP_093483067.1
Streptomyces ipomoeae	44.3	81%	5.00E-04	54.17	59	WP_141584693.1
Streptomyces sp. BV286	44.3	59%	5.00E-04	54.29	60	WP_217458224.1
Streptomyces	44.3	100%	6.00E-04	45.76	57	WP_010352623.1
Streptosporangium amethystogenes	44.3	96%	6.00E-04	43.86	57	WP_084195648.1
Frankia	44.3	59%	6.00E-04	57.14	61	WP_023842039.1
Streptomyces clavuligerus	44.7	94%	6.00E-04	37.7	77	WP_003953649.1
Streptomyces gossypiisoli	44.3	67%	6.00E-04	60	59	WP_180685958.1
Streptomyces tailanensis	44.3	89%	6.00E-04	50.94	59	WP_149829315.1
Streptomyces tsukubensis NRRL18488	47	81%	6.00E-04	52.08	226	EIF91476.1
unclassified Frankia	44.7	61%	6.00E-04	55.56	73	WP_011436256.1
Streptomyces	44.3	69%	6.00E-04	60.98	55	WP_093840813.1
Streptomyces sp. NRRL F-5065	44.3	89%	6.00E-04	49.06	59	WP_030422672.1
Streptacidiphilus sp. 4-A2	44.3	86%	6.00E-04	48.08	58	MBC3842095.1
Streptomyces kasugaensis	44.3	86%	7.00E-04	49.02	58	WP_094794154.1
Frankia	44.3	61%	7.00E-04	52.78	64	WP_023841893.1
Streptomyces sp. ISID311	43.9	94%	7.00E-04	50	58	WP_147245125.1
Streptomyces bathyalis	43.9	100%	7.00E-04	43.33	58	QPP10299.1
Pseudonocardiaceae bacterium	43.9	83%	7.00E-04	46.94	55	MPZ64707.1
Streptomyces lydicamycinicus	43.9	100%	7.00E-04	47.54	57	WP_078885789.1
Nocardiopsis metallicus	43.9	86%	7.00E-04	47.06	53	WP_184365798.1
Streptomyces	43.9	67%	7.00E-04	60	59	WP_053212995.1

Antribacter sp. KLBMP9083	44.3	83%	7.00E-04	46.94	69	WP_236089868.1
Streptomyces acidicola	43.9	67%	8.00E-04	60	59	WP_152860958.1
Streptomyces sp. CBMA29	43.9	79%	8.00E-04	55.32	58	WP_188284519.1
Streptomyces himalayensis	43.9	67%	8.00E-04	60	59	WP_181657648.1
Actinomadura craniellae	43.9	66%	8.00E-04	48.72	62	WP_111865174.1
Streptomyces	43.9	67%	8.00E-04	60	59	WP_030661977.1
unclassified Streptomyces	43.9	71%	8.00E-04	54.76	59	WP_093798518.1
Streptomyces olivaceus	43.9	79%	8.00E-04	53.19	59	WP_224295499.1
Streptomyces guanduensis	43.9	86%	9.00E-04	52.94	57	WP_093783313.1
Micromonospora sp. HM134	43.9	66%	0.001	51.28	64	WP_145923113.1
Verrucosispora sonchi	43.9	66%	0.001	51.28	64	WP_189041652.1
Streptomyces sp. GbtcB7	43.5	79%	0.001	55.32	57	WP_217570664.1
Verrucosispora sonchi	43.9	66%	0.001	51.28	67	GGM29362.1
Streptomyces bathyalis	43.9	86%	0.001	48.08	62	WP_246530589.1
Streptomyces sp. LS1784	43.5	81%	0.001	47.92	56	WP_224283691.1
Streptomyces	43.5	72%	0.001	52.27	60	WP_099506684.1
Allostreptomyces psammosilenae	43.5	100%	0.001	45.9	59	WP_179813698.1
Streptomyces viridochromogenes	43.5	67%	0.001	60	59	WP_003992136.1
Frankia sp. EUN1f	43.5	61%	0.001	50	64	WP_006541249.1
unclassified Kitasatospora	43.5	83%	0.001	51.02	54	WP_082527239.1
Streptomyces sp. NRRL S-920	43.5	61%	0.001	50	63	WP_030777606.1
Saccharomonospora marina	43.5	69%	0.001	57.14	61	WP_009156855.1
unclassified Streptomyces	43.5	55%	0.001	57.58	61	WP_219806425.1
Thermobifida halotolerans	43.5	74%	0.001	47.83	55	WP_084012818.1
unclassified Streptomyces	43.5	77%	0.001	54.35	59	WP_093740234.1
Streptomyces rubradiris	43.1	100%	0.001	44.07	57	WP_189998221.1
Nocardiopsis sp. MT53	43.1	64%	0.001	55.26	50	WP_220561791.1
Streptomyces sp. GS7	43.5	57%	0.001	52.94	63	WP_159509172.1
Nocardiopsis metallicus	43.1	62%	0.002	56.76	48	WP_184365133.1
Frankia sp. BMG5.36	43.1	86%	0.002	47.06	53	WP_241841609.1
Streptomyces glauciniger	43.1	67%	0.002	62.5	60	WP_089227175.1
Actinomycetia bacterium	43.1	67%	0.002	60	52	MBI3685783.1
Streptomyces sp. S816	42.7	84%	0.002	48	51	WP_136100964.1
Streptomyces sp. SN-593	43.1	67%	0.002	60	56	WP_202233258.1
Nocardioides speluncae	45.8	100%	0.002	49.15	255	WP_206050791.1
Streptomyces rubellomurinus	42.7	71%	0.002	53.49	55	WP_078860513.1
Amycolatopsis acididurans	42.7	72%	0.002	51.16	56	NKQ57287.1
Streptomyces sp. WZ.A104	42.7	86%	0.002	50.98	57	WP_096624476.1
Amycolatopsis acididurans	42.7	72%	0.002	51.16	52	WP_210718112.1

unclassified Crossiella	42.7	69%	0.002	52.08	61	WP_247763141.1
Allocatelliglobosispora scoriae	42.7	77%	0.002	47.83	51	MBB5871914.1
Nocardiopsis alba	42.4	67%	0.002	52.5	48	WP_014913548.1
Dactylosporangium sp.	42.7	76%	0.003	48.89	54	MBT8228016.1
Haloechinothrix halophila	42.7	77%	0.003	47.83	54	WP_231104725.1
unclassified Streptomyces	42.7	71%	0.003	52.38	59	WP_088575405.1
Streptomyces sp. CB01881	42.4	88%	0.003	51.85	56	WP_148641257.1
Pseudonocardiaceae	42.4	69%	0.003	54.76	61	WP_009151814.1
unclassified Streptomyces	42.4	67%	0.003	57.5	59	WP_171116581.1
Micromonospora echinospora	42.7	66%	0.003	48.72	68	WP_197701744.1
Thermobifida	42.4	76%	0.003	46.81	55	WP_083948129.1
Actinokineospora spheciospongiae	42.4	83%	0.003	40.82	57	WP_084175192.1
Kitasatospora xanthocidica	42.4	81%	0.003	50	56	WP_189923022.1
Micromonospora pallida	42.7	66%	0.003	48.72	68	SCL20318.1
Kribbella albertanoniae	42.4	74%	0.003	53.33	56	WP_132399725.1
Micromonospora pallida	42.4	66%	0.003	48.72	65	WP_091652553.1
Saccharomonospora amisosensis	43.5	66%	0.004	60	104	WP_208415804.1
Micromonospora echinospora	42.4	66%	0.004	48.72	65	WP_088985449.1
Phytomonospora endophytica	42.4	59%	0.004	51.43	56	WP_184790489.1
Streptomyces	42.4	98%	0.004	48.28	55	WP_078649233.1
Streptomyces	42.4	71%	0.004	52.38	59	WP_028444064.1
Streptomyces sp. PBSH9	42.4	71%	0.004	52.38	59	WP_243315508.1
Actinomadura pelletieri	42	71%	0.004	50	50	WP_121438342.1
Haloactinospora alba	42	55%	0.004	54.55	49	WP_141924148.1
Streptacidiphilus neutrinimicus	42	64%	0.004	57.89	54	WP_084729400.1
Natronosporangium hydrolyticum	42.4	86%	0.004	39.22	65	WP_239674899.1
Pseudonocardiaceae	42	69%	0.004	54.76	57	WP_083840819.1
Streptomyces globisporus	42	71%	0.004	52.38	59	WP_118898973.1
Amycolatopsis niigatensis	42.4	69%	0.004	54.76	61	WP_101434360.1
Micromonospora sp. Llam0	42.4	64%	0.004	47.37	66	WP_123606977.1
Micromonospora inyonensis	42.4	66%	0.004	48.72	68	SCL24718.1
Micromonospora inyonensis	42.4	66%	0.004	48.72	65	WP_091463422.1
Streptomyces sp. SBT349	42	55%	0.005	63.64	52	WP_234320330.1
Streptomyces luteolifulvus	42	59%	0.005	51.43	60	WP_150957501.1

```
NISIN               QNEFLLSYLPDIQKIVA--NLGGNLFFLRYTDP--KPHIRLRIKCSDLFLA-YGSIL--- 800
GALLIDERMIN         QDNFIRDYLFPFITELKVNKHINEFFYIKFKKD--EDFIKLRLFREDEDY---SQIYPFI 779
STREPTIN            QEELLNNWKKFWLNLPSELFSQSNMFYMRYNDT--NDHIRIRINCDSIENN-FSLYLSVV 792
PINENSIN            DRVLKEIVHPVIEDIQYKLKKTVKFFFIRYFEN--GYHIRLRLLLSSKEVSLFHSLLTYY 74
PAENILAN            ETDLVGLSLGHLLKQPGFKDWSRKFYFLRYVDP--EHHIRLRFQGEPDKLW--TAGLAQL 873
PENISIN             ADAFIGGPLLEFCRQAEEQGWAAGSFFMRYADP--DPHLRWRFCGHPEQLH--TVLLPQL 832
MICROBISPORICIN     QDDFLRDQVPVLVRAGIEH-GADRWFFIRYSDT-AGQHLRVRFRGEREKLW--AGLLPEI 886
PAENICIDIN          QNEFIGGYWEEFVRKHQDNGTILQAYFIRYADP--DKHLRVRFELSKNRTA--DGFLSTF 851
PAENIBACILLIN       LTDLLGSAFKTFMDNSA-EYFIDQVFFVQYADK--EPHIRLRCKMSGSVEHQRFTALNYV 830
SUBTILOMYCIN        ENEFIADELGSFIKKHLDKGTIKSSFFIRYSDP--KQHLRVRFKVEKNNIF--DTVLPII 831
FLAVUCIN            -DGFRE---RVIRRLFSTEETAAGAYFIRYPTDSGESGLRLRYPRRTSTG-------AAV 296
                       :                     :::::        :: *

NISIN               -EILKRSRKNRIMSTFDISIYDQEVERYGGFDTLELSEAIFCADSKIIPNLLTLIKDTNN 859
GALLIDERMIN         KKWKDYCLLNSELNDYSIVDYIPEVYRYGGPHVIENVEKFFMYDSLLSINIIQT----EF 835
STREPTIN            QDLLPQLIESCIISDIEVSSYKPEVNRYGGPHLISYAEEIFCKESILFLNNTIEFSDDE- 851
PINENSIN            ------ISDSDIKIILKEAQYIPETERYGNSDTIVYAENQFYASSRFVLNQLTESVPLTG 128
PAENILAN            NQWAAELREEGLITNMVIDTYHPELERYGGQTLMELAEQVFDVDSQWVIAYMKGIRSNAF 933
PENISIN             YRFTRQCVEDGLLSRLVIDTYDPEIERYGGRTLIGTAERLFCVDSRVVAEWIHARQSGHL 892
MICROBISPORICIN     GARLVEWQRQGLLAGHELGQYDPEYERYGGDALAEFTETAFQHDSAAAISLLRLTRRAGF 946
PAENICIDIN          HKWTEMLLHEGMIARVVIDTYEPEIERYGGSKPMAWAERMFSCDSEVTAKLVHLIRFNQV 911
PAENIBACILLIN       FDFLNTVTSSGLVSSYSVVPYRPEILRYGGAAFIEQAERLFAIDSYLVSGYYAGKRSINA 890
SUBTILOMYCIN        IKWLEEMRTKGFVNYYCFDPYNPEIERYGGEQSYKIAEDLFFCDSLIVSEWIQINRSGNV 891
FLAVUCIN            ETAIREMTSSGWLLGLEEKAHSLEFERYGGPAVFPLFAELFSLESRVVAHLSAASSTDTI 356
                             .          :  *  ***.          *  .*

NISIN               IK---NLFLKMIAQDFELQKVYSIIDSIIHVHNNRLIGIERDKEKLIYYTLQRLFVSEEY 991
GALLIDERMIN         ---------KHTLQENLYTSRSRIIGSFIHMRCNRIFGINPEKEKFVLSIFNEITKTKKY 970
STREPTIN            LMSYMKEYNRFGGTNDIYNTKFNLVGSFLHLSMNRLNGIDREFEEKVYCFAYYTLNAQQY 984
PINENSIN            NKKYT-------DSHLPAEAIDEALLSFIHMTNNRI-GIVNSEESYLLFLVRQTITLIKD 279
PAENILAN            IQQYAQAIILAKNNHELTNHPDDILGSIIHMHLNRLLGVNREREIKCMALAKHTVANLLH 1075
PENSIN              IARYVEGIRLCQAEGTLTNTQEDLMQSVIHMHLNRLFGIQREEEQKVLTLVRHTLYSLQH 1034
MICROBISPORICIN     VRRFGTAYREAFRPTDSPSTQLRLVGSLLHMTCNRLIGGSAERERSVLGLARGAVQDNLN 1109
PAENICIDIN          VKQYSHVLYEHETGGLSTNTREDIVFSVIHMHLNRLLGIDRDRERKIMIMARHALNSLVQ 1052
PAENIBACILLIN       LWQYAAYFTAIREADDRTNYYDDIMFSIMHMFCNRVFGLDREKEELALHLCYYSLEDYLQ 1019
SUBTILOMYCIN        AITYSKSIEN-----NPDFLIKNIIMSVIHMHCNRLYGINRDKENLVIGLAYHTIKNYNN 1025
FLAVUCIN            IRAHMKSLANKISAH---SVGTYELQSAAHLFCNRV-GISTDREKALWAALSKIER---- 470
                                            : *  *:  **: *   . *
```

Figure S9. Multiple sequence alignment for representative GLs associated with a select number of characterized lanthipeptides. All these GLs appear to be *anti*-GLs based on the conservation of key residues. Yellow = conserved Tyr in *anti*-GLs or conserved Lys in *syn*-GLs. Pink = conserved Arg residues that bind γ-carboxylate of glutamylated Ser/Thr. Blue = conserved Glu found across class I GLs. Green = conserved Arg in *anti*-GLs that acidifies the α-proton of glutamylated Ser/Thr residues for elimination; this Arg is substituted with a conserved Leu or Ala in *syn*-GLs. Red = conserved His for *anti*-GLs.

References

1. Sarksian, R.; Hegemann, J. D.; Simon, M. A.; Acedo, J. Z.; van der Donk, W. A., Unexpected methyllanthionine stereochemistry in the morphogenetic lanthipeptide SapT. *J. Am. Chem. Soc.* **2022**, *144* (14), 6373-6382.
2. Bothwell, I. R.; Caetano, T.; Sarksian, R.; Mendo, S.; van der Donk, W. A., Structural analysis of class I lanthipeptides from *Pedobacter lusitanus* NL19 reveals an unusual ring pattern. *ACS Chem. Biol.* **2021**, *16* (6), 1019-1029.
3. Acedo, J. Z.; Bothwell, I. R.; An, L.; Trouth, A.; Frazier, C.; van der Donk, W. A., *O*-methyltransferase-mediated incorporation of a β-amino acid in lanthipeptides. *J. Am. Chem. Soc.* **2019**, *141* (42), 16790-16801.

4. Tang, W.; van der Donk, W. A., The sequence of the enterococcal cytolysin imparts unusual lanthionine stereochemistry. *Nat. Chem. Biol.* **2013**, *9* (3), 157-9.
5. Tang, W.; Jiménez-Osés, G.; Houk, K. N.; van der Donk, W. A., Substrate control in stereoselective lanthionine biosynthesis. *Nat. Chem.* **2015**, *7* (1), 57-64.
6. Burkhart, B. J.; Schwalen, C.; Mann, G.; Naismith, J. H.; Mitchell, D. A., YcaO-dependent posttranslational amide activation: biosynthesis, structure, and function. *Chem. Rev.* **2017**, *117* (8), 5389-5456.
